# Generation of anti-GD2 CAR macrophages from human pluripotent stem cells for cancer immunotherapies

**DOI:** 10.1016/j.stemcr.2022.12.012

**Published:** 2023-01-12

**Authors:** Jue Zhang, Sarah Webster, Bret Duffin, Matthew N. Bernstein, John Steill, Scott Swanson, Matthew H. Forsberg, Jennifer Bolin, Matthew E. Brown, Aditi Majumder, Christian M. Capitini, Ron Stewart, James A. Thomson, Igor I. Slukvin

**Affiliations:** 1 Morgridge Institute for Research, Madison, WI 53715, USA; 2 Department of Pediatrics, University of Wisconsin–Madison, Madison, WI 53792, USA; 3 Department of Surgery, University of Wisconsin–Madison, Madison, WI 53792, USA; 4 Wisconsin National Primate Research Center, University of Wisconsin–Madison, Madison, WI 53715, USA; 5 Carbone Cancer Center, University of Wisconsin–Madison, Madison 53705, WI, USA; 6 Department of Cell & Regenerative Biology, University of Wisconsin–Madison, Madison, WI 53706, USA; 7 Department of Pathology and Laboratory Medicine, University of Wisconsin–Madison, Madison, WI 53705, USA

**Keywords:** chimeric antigen receptor, CAR, GD2, macrophages, pluripotent stem cells, PSCs, hemogenic endothelium, neuroblastoma, melanoma, immunotherapy

## Abstract

Macrophages armed with chimeric antigen receptors (CARs) provide a potent new option for treating solid tumors. However, genetic engineering and scalable production of somatic macrophages remains significant challenges. Here, we used CRISPR-Cas9 gene editing methods to integrate an anti-GD2 CAR into the AAVS1 locus of human pluripotent stem cells (hPSCs). We then established a serum- and feeder-free differentiation protocol for generating CAR macrophages (CAR-Ms) through arterial endothelial-to-hematopoietic transition (EHT). CAR-M produced by this method displayed a potent cytotoxic activity against GD2-expressing neuroblastoma and melanoma *in vitro* and neuroblastoma *in vivo*. This study provides a new platform for the efficient generation of off-the-shelf CAR-Ms for antitumor immunotherapy.

## Highlights

• Generation of hPSCs with anti-GD2 CAR inserted at AAVS1 locus
• Efficient generation of anti-GD2 CAR-Ms from hPSCs through arterial HE
• Anti-GD2 CAR-Ms possess potent activity against GD2^+^ solid tumors

## Graphical abstract

**Figure:**
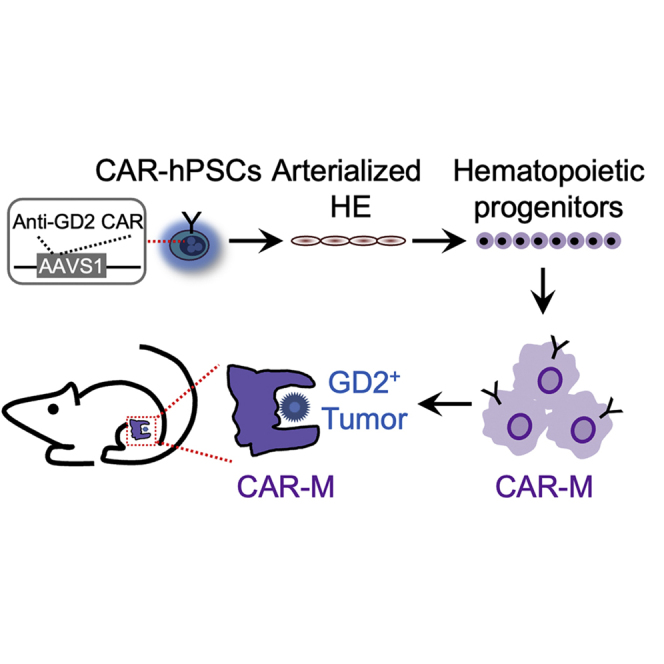

## Teaser

In this article, Zhang and colleagues show the generation of hPSCs with anti-GD2 CARs integrated into the AAVS1 locus and their differentiation into CAR macrophages (CAR-Ms) through arterialized hemogenic endothelium intermediates. CAR-Ms displayed a potent antitumor activity against neuroblastoma and melanoma.

## Introduction

Chimeric antigen receptor (CAR)-T cell and CAR-natural killer (NK) cell therapies have already demonstrated tremendous success in the eradication of lymphoid malignancies (Alcantara et al., 2018; Liu et al., 2020). However, many challenges remain in the application of CAR therapies for solid tumors (Klichinsky et al., 2020; Marofi et al., 2021a, 2021b), and responses with CAR-T cells have been limited to isolated, exceptional cases (Ahmed et al., 2015, 2017; Brown et al., 2016; Heczey et al., 2020; Hegde et al., 2017; Louis et al., 2011; Pule et al., 2008; Wang et al., 2018). Given the known capacity of macrophages to infiltrate and reside within solid tumors, CAR macrophages (CAR-Ms) have been proposed as a novel tool for immunotherapy of these tumors (Guedan et al., 2019; Klichinsky et al., 2020; Morrissey et al., 2018; Zhang et al., 2020). Despite that, generating macrophages from somatic cells represents a significant challenge, and transduction of monocytes/macrophages with lentiviruses is inefficient. While monocytes/macrophages can be transduced efficiently with adenoviral vectors, these vectors do not integrate into genomes and may not sustain prolonged CAR expression. Human pluripotent stem cells (hPSCs) are a logical alternative for large-scale production of CAR-Ms of uniform quality for off-the-shelf immunotherapy.

As a CAR target antigen, the disialoganglioside GD2 has been widely studied due to its high expression on solid tumors like neuroblastoma and melanoma, as well as documented improvement in survival with US Food and Drug Administration-approved anti-GD2 therapies like dinutuximab and naxitamab (Cheung et al., 2021; Yu et al., 2010). Since neuroblastoma is the most common and difficult-to-treat pediatric solid tumor, and melanoma remains the most lethal of the primary cutaneous neoplasms (Matthay et al., 2016; Switzer et al., 2022), we aim to generate hPSCs with an anti-GD2 CARs integrated into the AAVS1 locus and then differentiate them into macrophages by applying our arterial endothelium differentiation platform.

Using a *EFNB2-tdTomato/EPHB4-EGFP* dual-reporter hPSC line, we previously identified factors that regulate arterial endothelial cell specification and developed a method for the efficient generation of arterial endothelium in xeno-free, fully defined conditions (Zhang et al., 2017). In this study, we demonstrated that reducing cell-cell contact via low-density cell seeding produced arterialized endothelial cells with the capacity to undergo endothelial-to-hematopoietic transition (EHT), leading to the formation of blood cells. As we previously reported, arterial hemogenic endothelium (HE) is enriched in definitive lympho-myeloid progenitors, while nonarterial HE possesses mostly myeloid potential (Jung et al., 2021; Uenishi et al., 2018). In the present study, we created a new hPSC-derived arterial HE platform to generate macrophages expressing anti-GD2 CAR, which displayed potent antitumor activity against neuroblastoma and melanoma *in vitro* and neuroblastoma *in vivo*.

## Results

### Generation of hematopoietic progenitors using a modified arterial endothelium differentiation protocol

**Figure 1:**
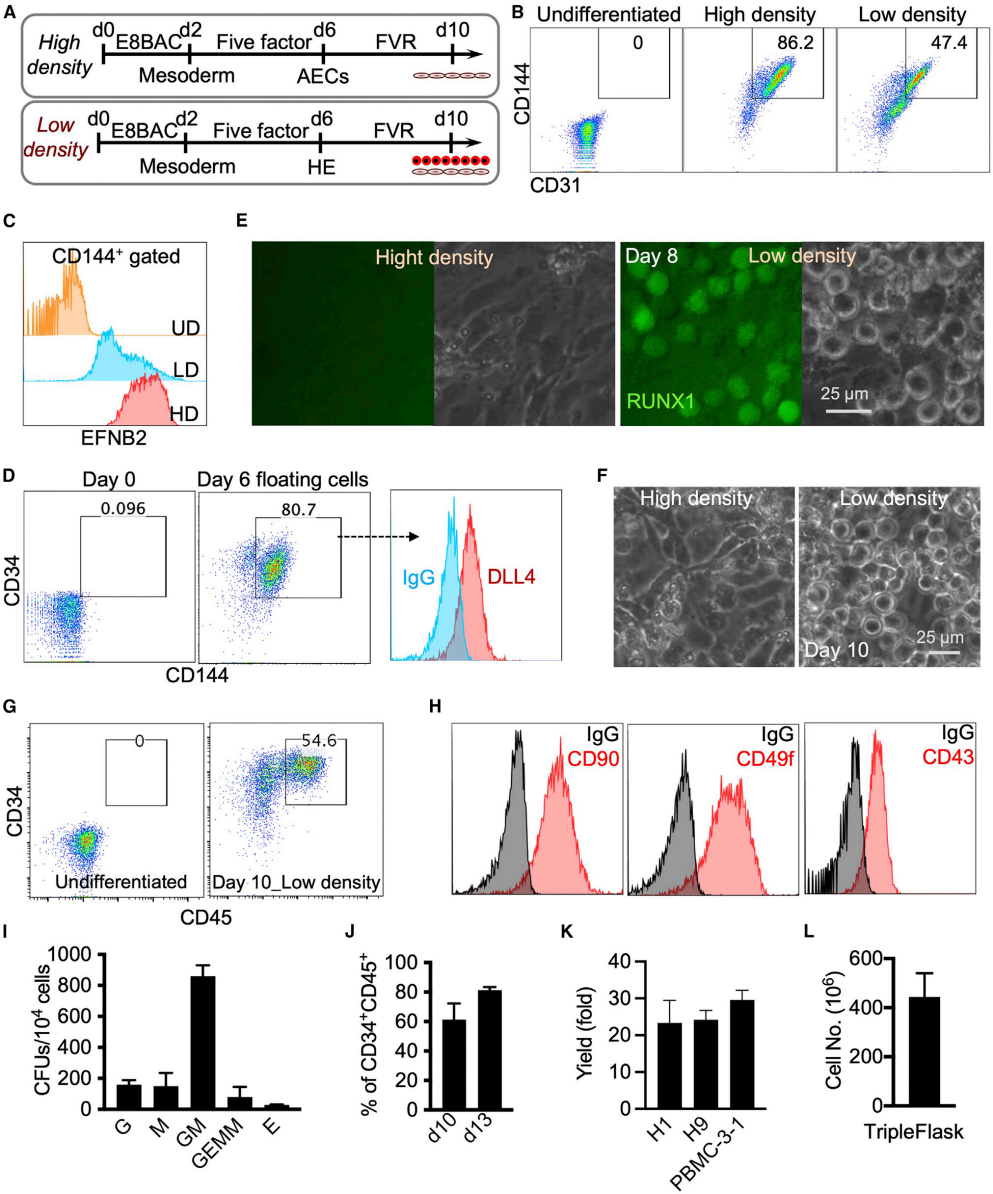
Low-density arterial endothelium differentiation cultures allow for the generation of blood cells H9 cells are used unless specified. (A) Schematic of arterial endothelial cell and hematopoietic cell differentiation. High cell density, 1.1 × 10^5^ cells/cm^2^. Low cell density, 1.8 × 10^4^ cells/cm^2^. FVR media contains FGF2, VEGFA, and RESV. (B) Representative flow cytometry dot plots show expression of CD31 and CD144 at day 6 of differentiation in low- and high-density conditions. Undifferentiated cells (day 0) are used as a negative control. (C) CD144^+^ cells in low density (LD) and high density (HD) were gated for analysis of EFNB2 expression. Undifferentiated cells (UDs) are used as a negative control. The *EFNB2-tdTomato/EPHB4-EGFP* H1 reporter cell line was used. (D) Representative flow cytometry analysis of CD144, CD34, and DLL4 expression of floating cells collected at day 6 of differentiation. (E) RUNX1+23 enhancer activity in LD and HD cultures at day 8 of differentiation. The RUNX1+23 enhancer-Venus reporter cell line was used. (F) Phase contrast images of arterial endothelium cultures at day 10 of differentiation. (G) Representative flow cytometry analysis of CD34 and CD45 expression at day 10 of differentiation. (H) Representative flow cytometry analysis of CD90, CD49f, and CD43 expression of floating cells collected at day 10 of differentiation. (I) Colony-forming unit assay of day 8 cells in low cell density condition. Data are represented as mean ± SD; n = 3 independent experiments. (J) Percentages of CD34^+^CD45^+^ cells at days 10 and 13 of differentiation. Data are represented as mean ± SD. Student’s t test; ^∗^p < 0.05; n = 3 independent experiments. (K) Hematopoietic cells generated from one starting hPSC. H1 and H9 embryonic stem cells and PBMC-3-1 hiPSCs were used. Data are represented as mean ± SD; n = 3 independent experiments. (L) Total floating hematopoietic cell number generated from one T500 TrypleFlask. Data are represented as mean ± SD; n = 3 independent experiments.

We have previously established a highly efficient, fully defined, xeno-free protocol for differentiation of arterial endothelial cells from hPSCs (Zhang et al., 2017). However, this protocol did not lead to blood formation where arterial endothelial cell differentiation was performed with hPSCs seeded at high cell density (Figure 1A) (Zhang et al., 2017). Since the strengthening of tight junctions between aortic endothelial cells in high-density cultures may repress specification of HE and subsequent EHT (Zhang et al., 2014), we hypothesized that reducing cell-cell contact by seeding the cells at low density may allow for the generation of arterial endothelium with hemogenic potential. To test this hypothesis, we used differentiation conditions and media similar to our prior protocol but decreased the initial cell density to 1.8 × 10^4^ versus 1.1 × 10^5^ cells/cm^2^ density in our original protocol (Figure 1A). Decreasing the cell density reduced the proportion of CD31^+^CD144^+^ endothelial cells in day 6 differentiation cultures (Figure 1B). However, these endothelial cells still retained arterial fate as evidenced by EFNB2 expression (Figure 1C). Additionally, a few rounds, floating cells were observed on day 6 of differentiation in the low-density protocol. These cells co-expressed CD144 and CD34, as well as moderate levels of DLL4 (Figure 1D), consistent with their origin from arterial HE.

To further induce hematopoiesis, the medium was then switched to fibroblast growth factor 2 (FGF2), vascular growth factor A (VEGFA), and resveratrol (RESV) containing medium (FVR medium), and cells were cultured for 4 more days (Figure 1A). Using the RUNX1+23-Venus enhancer-reporter H1 hPSC line (Uenishi et al., 2018), we observed that round, nonadherent, RUNX1+23^+^ blood cells formed at day 8 of differentiation in low-density, but not in high-density, conditions (Figure 1E). At day 10, more floating hematopoietic cells expressing CD34 and CD45 were observed in the cultures (Figures 1F and 1G). These hematopoietic cells also expressed CD90, CD49f, and CD43 (Figure 1H) and displayed multilineage differentiation potential via a colony-forming unit (CFU) assay (Figure 1I). The numbers and percentages of CD34^+^CD45^+^ cells could be further increased up to 80% purity after culture for an additional 3 days (Figure 1J). This protocol produced more than 20 hematopoietic progenitors from one starting hPSC from various pluripotent cell lines (H1, H9, and PBMC-3-1) and routinely generated 4 × 10^8^ hematopoietic progenitors from a single T500 TripleFlask (Figures 1K and 1L). Together, these data demonstrate that reducing cell-cell contact by seeding cells at low density supports formation of arterial HE with the capacity to undergo EHT and formation of CD34^+^ hematopoietic progenitors expressing DLL4.

### Generation and characterization of CAR-Ms

**Figure 2:**
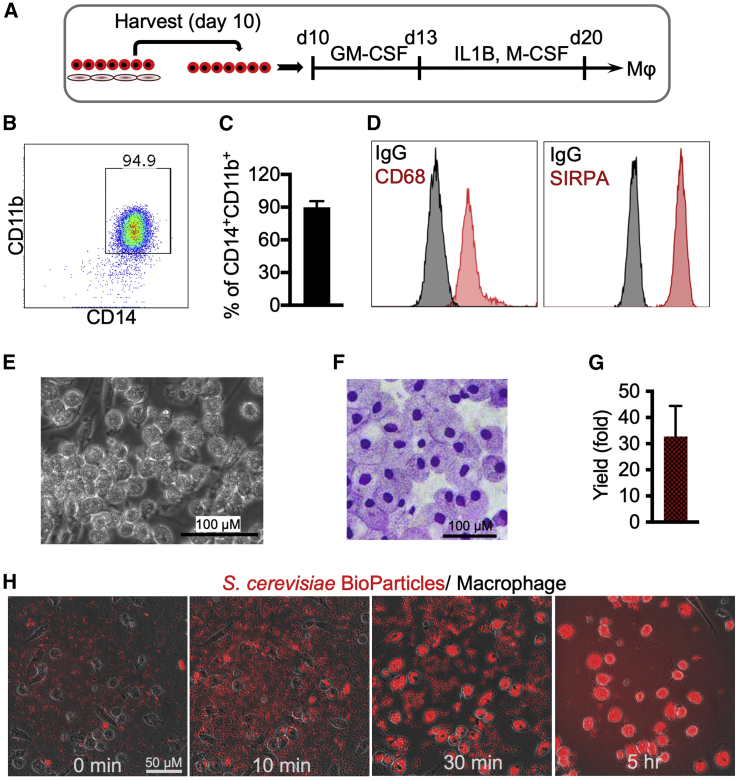
Generation and functional assessment of macrophages generated from H9 hPSCs (A) Schematic of macrophage differentiation. Hematopoietic cells on day 10 of differentiation are collected and cultured in serum-free media containing GM-CSF for 3 days and then with IL-1β and M-CSF for another 7 days. (B) Representative dot plot shows flow cytometry analysis of CD14 and CD11b expression at day 20 of differentiation. (C) Percentages of CD14^+^CD11b^+^ macrophages are presented as mean ± SD, n = 3 independent experiments. (D) Representative histograms show flow cytometry analysis of CD68 and SIRPA/CD172A expression at day 20 of differentiation. (E) Cell morphology at day 20 of differentiation. (F) Wright-Giemsa staining of cytospins from day 20 differentiation. (G) Total yield of macrophages from one starting hPSC. Data are represented as mean ± SD, n = 3 independent experiments. (H) Phagocytosis of yeast particles by macrophages. Zymosan An *S*. *cerevisiae* BioParticles (Texas Red conjugate; Life Technologies) were prepared in phosphate-buffered saline (PBS; 10 mg/mL = 2 × 10^9^ particle/mL). 20 μL particles were added to 2 mL media containing 4 × 10^5^ macrophage. Phagocytosis was imaged over time.

To generate macrophages using our optimized arterial endothelium differentiation protocol, we collected the hematopoietic cells on day 10 differentiation and cultured them in serum-free media containing granulocyte-macrophage colony-stimulating factor (GM-CSF) for 3 days and then interleukin-1β (IL-1β) and macrophage CSF (M-CSF) for another 7 days (Figure 2A). The protocol generated ∼90% of CD14^+^CD11b^+^ macrophages, which also expressed CD68 and SIRPA/CD172A (Figures 2B–2D). In addition, they were large in size (20 μm diameter) and possessed typical macrophage morphology (Figures 2E and 2F). The final yield of macrophages was approximately 30-fold from the starting hPSCs (Figure 2G).

To characterize the phagocytotic function of the macrophages, BioParticles were added to macrophage cultures. The results revealed that these macrophages were functional and able to uptake yeast particles (Figure 2H; Video S1).

Video S1. Phagocytosis yeast particles Phagocytosis of yeast particles (red) by WT-M for one day.

**Figure 3:**
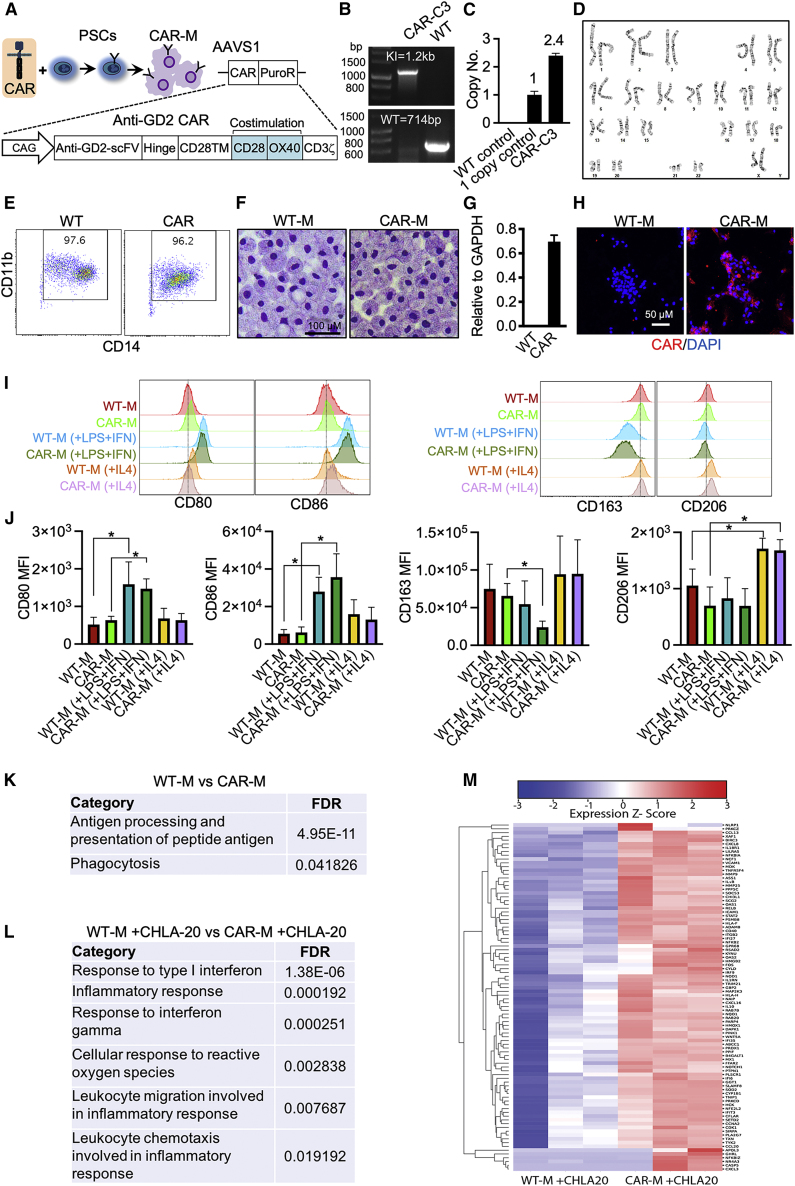
Engineering anti-GD2 CAR hPSCs and functional analysis of anti-GD2 CAR-Ms (H9 derived) (A) Schematic of CAR constructs and CAR-engineered cells. CAG, CMV enhancer/chicken β action promoter; scFv, single chain fragment variable; TM, transmembrane. (B) Junctional PCR analysis AAVS1-CAR knockin (KI) allele and WT AAVS1 allele to demonstrate a correct CAR integration. WT cells (without gene editing) are used as a control. (C) qPCR analysis of AAVS1-GD2-CAR-PuroR copy number. Data are represented as mean ± SD. n = 2 independent experiments. (D) Karyotyping of CAR-C3 hPSC line. (E) Flow cytometry analysis of CD14 and CD11b expression of macrophages. (F) Wright-Giemsa staining of WT-M and CAR-M cytospins. (G) qRT-PCR analysis of CAR expression in macrophages. n = 3 independent experiments. (H) Demonstration of anti-GD2 CAR expression in macrophages using immunofluorescence staining with antibody against GD2 antibody 14G2a. (I) Representative flow cytometry plots show expression of M1/M2 markers in WT-Ms and CAR-Ms with or without treatment. (J) Statistics of M1/M2 markers. MFI, mean fluorescent intensity. Data are presented as mean ± SD. Student’s t test; ^∗^p < 0.05; n = 4 independent experiments. CAR-Ms are generated from 2 different clones. (K) M1-related GO terms enriched in CAR-Ms. (L) M1-related GO terms enriched in CAR-Ms co-cultured with CHLA-20 cells. (M) Heatmap shows M1-related genes in macrophages co-cultured with CHLA-20.

To generate macrophages with antigen-dependent antitumor potential, we applied CRISPR-Cas9 technology to knock in a third-generation anti-GD2 CAR (14G2a-CD28-OX40-CD3ζ) into the AAVS1 locus of H9 hPSCs (Figures 3A–3D). GD2-CAR hPSCs were then differentiated into anti-GD2 CAR-Ms (Figure 3E), which displayed similar morphology with wild-type macrophages (WT-Ms) (Figure 3F). qRT-PCR and immunostaining confirmed the expression of anti-GD2 CAR in CAR-Ms (Figures 3G and 3H). Macrophages can be polarized into M1 (antitumor, pro-inflammatory) and M2 (protumor, antiinflammatory) macrophages (Pan et al., 2020). To characterize the M1/M2 phenotypes, we treated macrophages with lipopolysaccharide (LPS) and interferon γ (IFNγ) to induce M1 polarization or IL-4 to induce M2 polarization. The results revealed that CD80 and CD86 (M1 markers) expression was greatly increased by LPS and IFNγ in both WT-Ms and CAR-Ms (Figures 3I and 3J). In addition, LPS and IFNγ decreased CD163 expression (an M2 marker) in CAR-Ms but not WT-Ms (Figures 3I and 3J). On the other hand, IL-4 increased CD206 expression (an M2 marker) in both WT-Ms and CAR-Ms (Figures 3I and 3J).

To further characterize the CAR-Ms, we performed bulk RNA sequencing of WT-Ms and CAR-Ms collected from differentiation cultures and from co-cultures with GD2-expressing CHLA-20 neuroblastoma. Gene Ontology (GO) analysis of differentially expressed genes in WT-Ms and CAR-Ms before co-culture with tumor cells revealed that only two M1-related pathways, antigen processing and presentation of peptide antigen and phagocytosis, were enriched in CAR-Ms when compared with WT-Ms (Figure 3K). After co-culture with CHLA-20 tumor cells, more M1-related pathways were enriched in CAR-Ms, namely response to type I IFN, inflammatory response, response to IFNγ, cellular response to reactive oxygen species, leukocyte migration involved in inflammatory response, and leukocyte chemotaxis involved in inflammatory response (Figure 3L). Heatmap analysis also revealed the upregulation of numerous M1-related genes in CAR-Ms co-cultured with CHLA-20 (Figure 3M). These results suggest that CAR expression in hPSC-derived macrophages promotes their polarization toward an M1 state after co-culture with tumor cells.

### Antitumor activity of CAR-Ms

**Figure 4:**
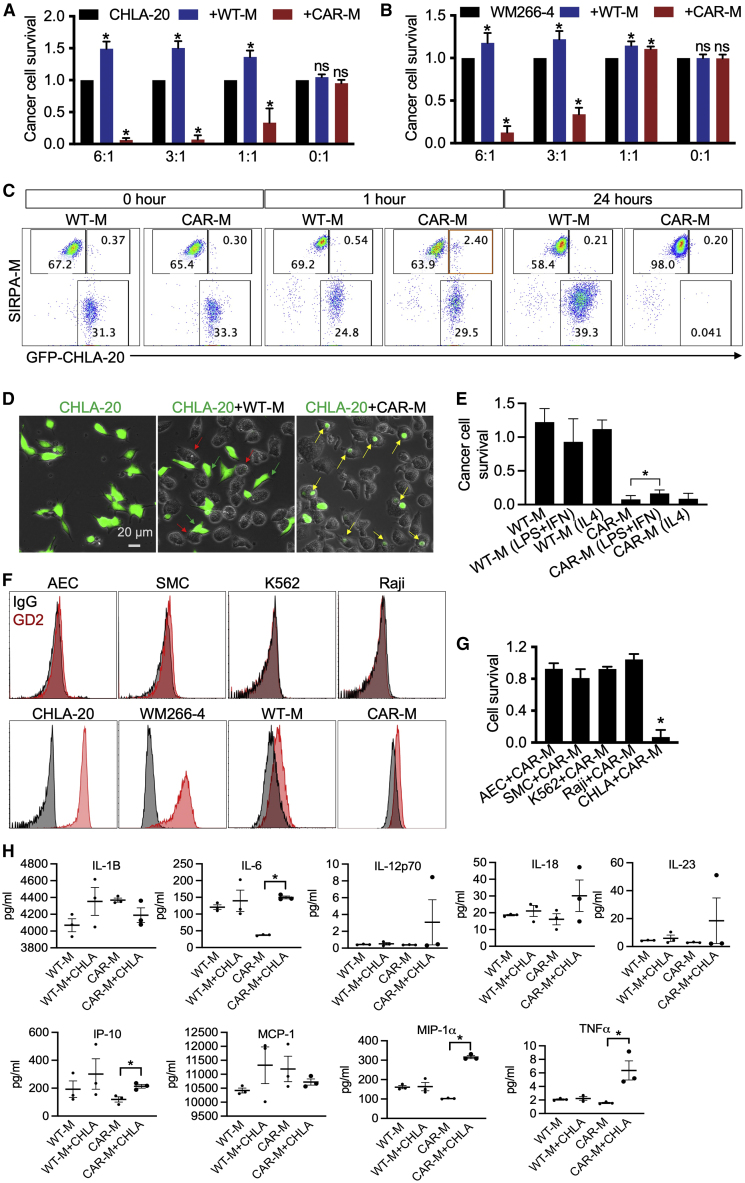
Evaluation of antitumor activity of CAR-Ms (H9 derived) *in vitro* (A) Killing of CHLA-20 neuroblastoma cells by CAR-Ms. CHLA-20-AkaLuc-GFP cells were mono-cultured or co-cultured with macrophages at different E:T ratios for 20–24 h. Statistics of CHLA-20 cell survival results are represented as mean ± SD. Student’s t test; ^∗^p < 0.05; ns, not significant; n > 4 independent experiments. (B) Killing of WM266-4 melanoma cells by CAR-Ms. WM266-4-AkaLuc-GFP melanoma cells were mono-cultured or co-cultured with macrophages at different E:T ratios for 20–24 h. Statistics of WM266-4 cell survival. Results are mean ± SD. Student’s t test; ^∗^p < 0.05; ns, not significant; n > 4 independent experiments. (C) Representative flow cytometry plots show CHLA-20 cells co-cultured with WT-Ms and CAR-Ms. CHLA-20 cells are labeled by GFP. WT-Ms and CAR-Ms are labeled by SIRPA immunostaining. (D) Representative images show phagocytosis of CHLA-20 cells by CAR-Ms. CHLA-20-AkaLuc-GFP cells were co-cultured with macrophages for 6 h (E:T = 3:1). Green arrows indicate CHLA-20 cells, red arrows indicate WT-Ms, and yellow arrows indicate phagocytosis of CAR-Ms. (E) Killing of CHLA-20 neuroblastoma cells by CAR-Ms with or without treatment. CHLA-20-AkaLuc-GFP cells were mono-cultured or co-cultured with macrophages at E:T ratio = 3:1 for 20–24 h. Statistics of CHLA-20 cell survival results are represented as mean ± SD. Student’s t test; ^∗^p < 0.05; n = 3 independent experiments. (F) Representative histograms show flow cytometry analysis of GD2 expression in different cells. AEC is aretrial endothelial cells, SMC is smooth muscle cells. (G) CAR-Ms were co-cultured with AEC-NOS3-NanoLuc-2A-tdTomato, SMC-MYH11-NanoLuc-2A-tdTomato, K562-AkaLuc-GFP, Raji-AkaLuc-GFP, and CHLA-20 cells (E:T = 3:1) for 20 h. Target cell survival was measured by luciferase assay. Results are represented as mean ± SD. Student’s t test; ^∗^p < 0.05; n = 3 independent experiments. (H) Secretome analysis of macrophages. WT-Ms and CAR-Ms were mono-cultured or co-cultured with CHLA-20 for 20 h. Cell culture media were collected for secretome analysis. Results are mean ± SD. Student’s t test; ^∗^p < 0.05; n = 3 independent experiments.

Next, we evaluated the antitumor activity of CAR-Ms *in vitro*. WT-Ms or CAR-Ms were co-cultured with CHLA-20-AkaLuc-GFP neuroblastoma cells or WM266-4-AkaLuc-GFP melanoma cells at different effector-to-target (E:T) ratios. CAR-Ms demonstrated significant antitumor activity at a 1:1 E:T ratio versus CHLA-20 cells using a luciferase-based cytotoxicity assay (Figure 4A). Antitumor activity was further improved with the increased E:T ratio (Figure 4A). CAR-Ms were also able to inhibit WM266-4 cell growth at a 3:1 or higher E:T ratio (Figure 4B). In contrast, WT-Ms showed no antitumor activity against neuroblastoma and melanoma cells and appeared to promote tumor growth (Figures 4A and 4B). Time-lapse results also demonstrated that CAR-Ms were able to eliminate CHLA-20-AkaLuc-GFP cells in 1 day (Videos S2 and S3).

Video S2. Phagocytosis cancer cells + WT macrophages CHLA-20-AkaLuc-GFP cells were co-cultured with WT-M for one day (E:T = 3:1).

Video S3. Phagocytosis cancer cells + CAR macrophages CHLA-20-AkaLuc-GFP cells were co-cultured with CAR-M for one day (E:T = 3:1).

To further explore the interaction between tumor cells (GFP labeled) and macrophages (SIRPA stained), we performed flow cytometric analysis of co-cultured cells at different time points (Figure 4C). At 24 h, CHLA-20 cells were not reduced by WT-Ms, while most of the CHLA-20 cells were eliminated by CAR-Ms (Figure 4C). The GFP^+^SIRPA^+^ cells were found at 1 h of CHLA-20 and CAR-M co-culture by flow cytometry (Figure 4C), indicating that CHLA-20 cells were engulfed by CAR-Ms. Fluorescence imaging further confirmed phagocytosis of CHLA-20 cells by CAR-Ms (Figure 4D, yellow arrows indicated).

To investigate whether the antitumor activity of CAR-Ms was dependent on the M1/M2 phenotype, we pre-treated WT-Ms and CAR-Ms with LPS and IFNγ or IL-4. Interestingly, although LPS and IFNγ were able to enhance M1 marker expression (Figures 3I and 3J), they failed to improve the antitumor activity of both WT-Ms and CAR-Ms (Figure 4E). Instead, the antitumor activity of CAR-Ms was slightly reduced (Figure 4E). IL-4 promoted M2 polarization (Figures 3I and 3J) but did not suppress the antitumor activity of CAR-Ms (Figure 4E). The results suggested that the antitumor activity of CAR-Ms was independent of the M1/M2 phenotype induced by LPS, IFNγ, and IL-4.

To assess the antitumor specificity, we co-cultured CAR-Ms with GD2^−^ allogeneic healthy cells: arterial endothelial cells (AEC-NOS3-NanoLuc-2A-tdTomato) and smooth muscle cells (SMC-MYH11-NanoLuc-2A-tdTomato), as well as GD2^−^ tumor cells: Raji-AkaLuc-GFP and K562-AkaLuc-GFP (Figures 4F and 4G). Luminescence results revealed that CAR-Ms had minimal activity against these cells (Figure 4G), demonstrating the antigen specificity of anti-GD2^+^ tumor activity. CAR-T cell therapy could trigger life-threatening cytokine-release syndrome (CRS), so we measured the cytokine release of macrophages before and after they were co-cultured with CHLA-20 cells. CRS-related cytokines can increase 30- to 8,000-fold upon CAR-T injection (Lee et al., 2019; Norelli et al., 2018). In our results, IL-6, IP-10, MIP-1α, and tumor necrosis factor α (TNF-α) were only increased 2- to 4-fold in CAR-Ms after co-culture with CHLA-20 (Figure 4H), indicating minimal risk of CRS from CAR-Ms.

To demonstrate that our CAR-M protocol is applicable to other hPSC lines, we generated another anit-GD2 CAR cell line from PBMC-3-1 human induced PSCs (hiPSCs) (Figures S1A–S1C) by using the same strategy shown in Figure 3. Anti-GD2 CAR hiPSCs differentiated into GD2-CAR-Ms and demonstrated antitumor activity against both CHLA-20 and WM266-4 cells (Figures S1D and S1E).

**Figure 5:**
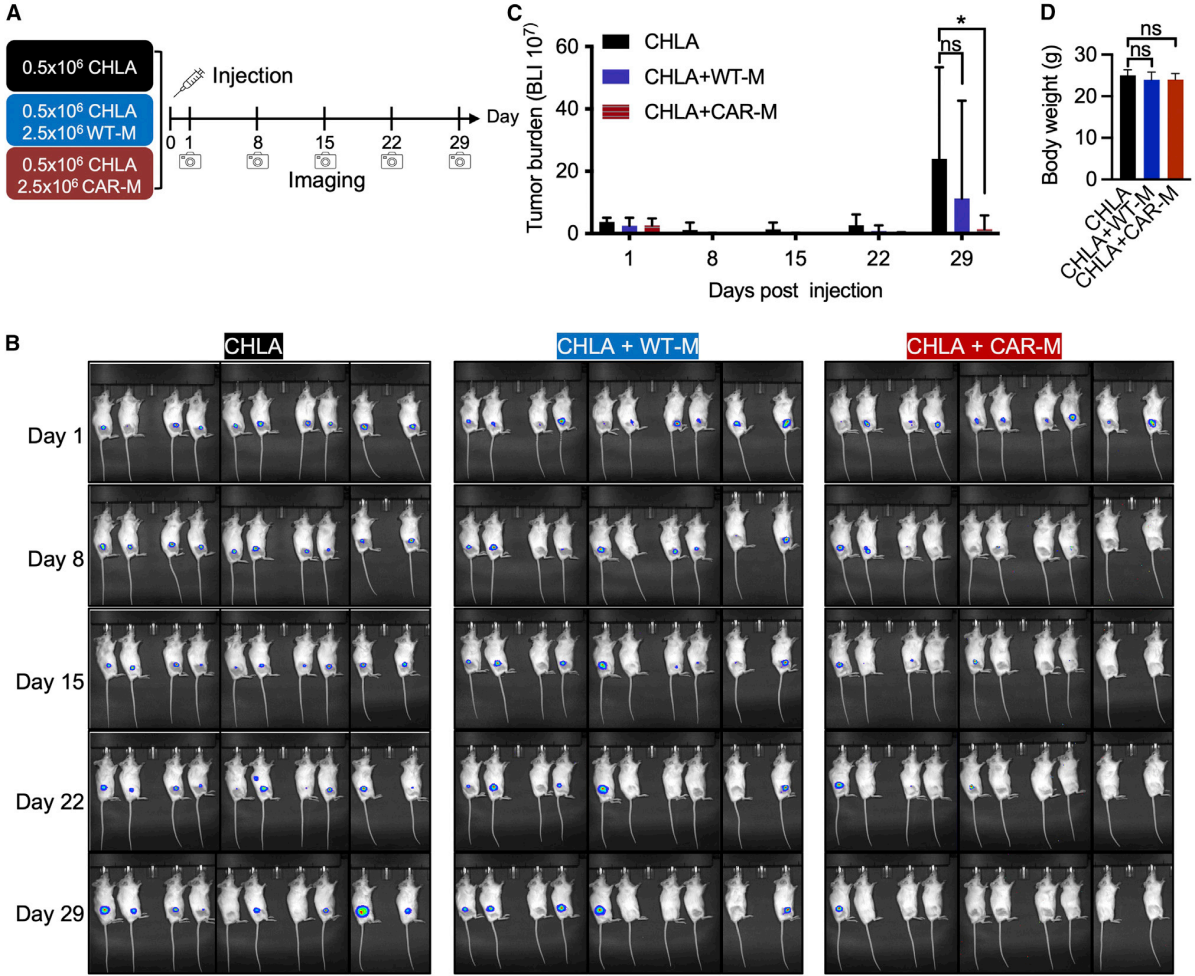
CAR-Ms (H9 derived) significantly reduce tumor burden in a CHLA-20 xenograft mouse model (A) Schematic of mouse model experiments. CHLA-20-AkaLuc-GFP cells were injected subcutaneously alone or with WT-Ms or CAR-Ms. Luminescent signals were measured at 1, 8, 15, 22, and 29 days post injection. 10 mice per group. (B) Tumor burden was assessed by bioluminescent imaging at indicated time points. (C) Quantification of tumor burden as shown by luminescent signals. Results are represented as mean ± SD. Student’s t test; ^∗^p < 0.05; ns, not significant; n = 10 mice. (D) Body weight represented as mean ± SD. Student’s t test; ns, not significant; n = 10 mice.

To verify antitumor activity *in vivo*, 5 × 10^5^ CHLA-20-AkaLuc-GFP cells were subcutaneously injected into the hind flank of NSG mice alone or with 2.5 × 10^6^ WT-M or CAR-M (H9 hPSC derived) (5:1 E:T ratio) (Figure 5A). These studies revealed that CAR-M-treated mice had significantly reduced tumor burden 4 weeks post injection as evidenced by 90% tumor-free mice with CAR-M treatment compared with 50% tumor-free mice with WT-M treatment (Figure 5B). Significant differences in tumor burden were observed between mice injected with CHLA-20 alone versus mice injected with both CHLA-20 and CAR-Ms, while no statistical differences in tumor burden were observed between mice injected with both CHLA-20 and WT-Ms versus mice with CHLA-20 alone (Figure 5C). CAR-M treatment did not reduce the body weight (Figure 5D), indicating minimal adverse effects of the cell therapy. H&E staining further showed the normal histology of heart, lung, liver, and spleen after CAR-M treatment (Figure S2).

We also tested the antitumor activity of hiPSC-derived CAR-Ms in mice injected with 1 × 10^6^ CHLA-20 and 4 × 10^6^ CAR-M (PBMC-3-1 hiPSC-derived) (4:1 E:T ratio). As shown in Figure S3, hiPSC-derived CAR-Ms significantly reduced tumor burden, while unmodified WT-Ms did not affect tumor growth. In summary, CAR-Ms generated from arterial HE demonstrated superior antitumor activity compared with WT-Ms.

## Discussion

Adoptive immunotherapies provide a promising therapeutic option for treating hematologic malignancies. CAR-T and CAR-NK cells represent frontrunners in this approach. Recently, macrophages were investigated for immunotherapy, and the administration of macrophages in patients with cancer has been proved to be well tolerated (Monnet et al., 2002). However, unmodified macrophages exhibited little antitumor activity. Thus, genetic modification of macrophages with CARs is a promising method to bolster their antitumor effects (Klichinsky et al., 2020; Zhang et al., 2020). In previous studies, enhanced antitumor activity was demonstrated in murine macrophages engineered to express CARs with FcRγ, Megf10, and CD3ζ signaling units (Morrissey et al., 2018) and human macrophages expressing the CD3ζ signaling unit (Klichinsky et al., 2020). hiPSC-derived macrophages have been engineered to express CD20-CAR with FcγR1 (Senju et al., 2011), CD19, or mesothelin CAR with CD3ζ signaling domains (Zhang et al., 2020), enabling them to destroy leukemic cells or ovarian cancers. However, the efficacy and persistency need to be further improved in hiPSC-derived CAR-Ms (Zhang et al., 2020).

Here, we demonstrated the utility of a scalable hPSC platform that reproducibly generates uniform CAR-Ms with anti-GD2-dependent antitumor activities through arterial HE. hPSC-derived anti-GD2 CAR-Ms demonstrated superior killing of neuroblastoma and melanoma cells *in vitro* and killing of neuroblastoma cells *in vivo*. To our knowledge, these data are the first evidence of CAR-Ms, hPSC or somatic cell derived, having *in vivo* activity against a pediatric solid tumor.

hPSC-based platforms for CAR-M production have several advantages over somatic myeloid cell-based platforms. Given that human hPSCs can be expanded almost indefinitely in culture, essentially an unlimited number of the desired therapeutic cells can be produced from an established hPSC line. Additionally, hPSCs can be subjected to multiplex editing approaches to introduce multiple genetic traits and clonally selected to ensure homogeneity of genetic editing. Such uniformity in multiplex gene editing would be difficult to achieve using primary somatic myeloid cells. hPSCs bearing anti-GD2 CARs can be further genetically modified to abrogate human leukocyte antigen (HLA) expression to provide hypoimmunogenic macrophages suitable for administration across HLA immunological barriers. In addition to CAR-Ms, the same gene-edited hPSC cell line could be used for generation of CAR-T or CAR-NK cells for combination immunotherapy.

In prior studies, CAR-Ms were generated via transduction with CAR-expressing lentiviruses (Zhang et al., 2020) or transfection with CAR-expressing plasmids (Senju et al., 2011). In the present study, we demonstrate that a CAR inserted into the safe-harbor AAVS1 locus in hPSCs by CRISPR-Cas9 avoids the gene silencing that commonly occurs with the above-mentioned methods following differentiation. Overcoming this obstacle in our platform allows for the stable CAR expression in hPSC-derived macrophages. Additionally, we develop an efficient serum- and feeder-free system for macrophage generation based on a modification of our previously established arterial endothelial differentiation protocol. Our anti-GD2 CAR-Ms demonstrate superior antitumor activities against neuroblastoma cells *in vitro* and *in vivo*. Neuroblastoma is the most common and difficult-to-treat type of pediatric solid tumor due to its high genetic heterogeneity (Boeva et al., 2017), making identification of “druggable” targets for small-molecule inhibitors difficult and generation of neoantigens for T cell-based immunotherapies ineffective. However, the neuroblastoma tumor microenvironment is enriched with macrophages (Frosch et al., 2021), and recent data indicate that macrophages could be important mediators of antitumor responses against neuroblastoma in the context of anti-GD2 (redirecting against the tumor) and anti-CD47 (blocking phagocytosis inhibitory pathway) dual-antibody therapy (Theruvath et al., 2022). Here, we show that CAR-Ms may provide an alternative antigen-specific cellular treatment for neuroblastoma. Having an established hPSC-based CAR-M therapy for solid tumors, we are able to explore next-generation cell therapy by introducing additional genetic modifications to enhance antitumor effects or by using a combination CAR-Ms and CAR-T or CAR-NK cell therapies.

## Experimental procedures

### Resource availability

#### Corresponding author

Further information and requests for resources and reagents should be directed to and will be fulfilled by the corresponding author, Dr. Igor I. Slukvin (islukvin@wisc.edu).

#### Materials availability

Anti-GD2 CAR plasmid and cell lines generated in this study will be made available on request, but we may require a payment and/or a completed materials transfer agreement if there is potential for commercial application.

### Macrophage differentiation

hPSCs were seeded at low density and differentiated into hematopoietic progenitors by using arterial endothelial cell differentiation media. The hematopoietic progenitors were further induced into macrophages by treatment with macrophage differentiation media.

### *In vitro* cytotoxicity assay

Macrophages were co-cultured with different cells. The target cell survival was measured by luciferase assay.

### *In vivo* antitumor activity analysis

The animal experiments were performed under approval from the UW–Madison Institutional Review Board. Neuroblastoma cells CHLA-20-AkaLuc-GFP and macrophages were injected into the hind flank of the mice. Antitumor effect was monitored by bioluminescent imaging using IVIS imaging system at the indicated time.

Please refer to the supplemental experimental procedures for more detailed methods.

### Ethics approval

The animal experiments were performed under approval from UW–Madison, Institutional Review Board.

## Author contributions

J.Z. designed and performed the experiments, analyzed the data, and wrote the paper. S.W. and B.D. performed cell culture. M. B., J.S., S.S., and R.S. analyzed RNA-seq data. M.F. performed secretome analysis. J.B. performed RNA-seq. M.B. revised the manuscript. A.M. performed cytospin experiment. C.M.C., J.A.T., and I.I.S. designed the experiments and revised the manuscript.

## Data Availability

NA sequencing (RNA-seq) data is available on Gene Expression Omnibus (GEO) database: GSE201174.

## Supplemental information

Document S1. Figures S1–S3, Tables S1–S4, and supplemental experimental procedures

Document S2. Article plus supplemental information
