# Supplementary material for: Generation of anti-GD2 CAR macrophages from human pluripotent stem cells for cancer immunotherapies
Source: Stem Cell Reports. 2023 Jan 12;18(2):585–96. doi: 10.1016/j.stemcr.2022.12.012 (PMC9968983; doi:10.1016/j.stemcr.2022.12.012)
Supplement: Document S1. Figures S1–S3, Tables S1–S4, and supplemental experimental procedures [file mmc1.pdf]

Stem Cell Reports, Volume *18*

Supplemental Information

Generation of anti-GD2 CAR macrophages from human pluripotent stem cells for cancer immunotherapies

Jue Zhang, Sarah Webster, Bret Duffin, Matthew N. Bernstein, John Steill, Scott Swanson, Matthew H. Forsberg, Jennifer Bolin, Matthew E. Brown, Aditi Majumder, Christian M. Capitini, Ron Stewart, James A. Thomson, and Igor I. Slukvin

Figure legends

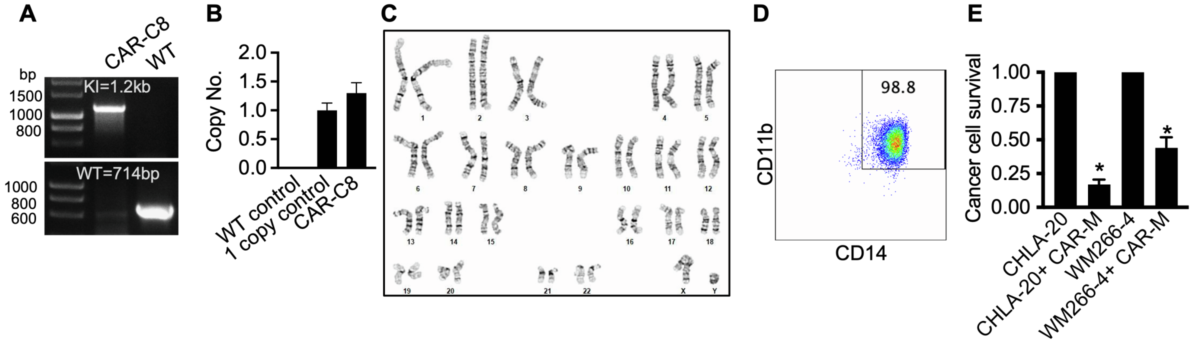

Figure S1. Generation of CAR-M from PBMC-3-1 hiPSCs. Related to Figure 3 and 4.

(A) Junctional PCR analysis AAVS1-CAR KI and WT allele to demonstrate a correct CAR integration.

(B) qPCR analysis of AAVS1-anti-GD2-CAR-PuroR copy number. Data are represented as mean ±SD; n = 3 independent experiments.

(C) Karyotyping of Anti-GD2 CAR hiPSCs.

(D) Representative dot plot shows flow cytometry analysis of CD14 and CD11b expression at day 21 of differentiation.

(E) Killing of CHLA-20 neuroblastoma and WM266-4 melanoma cells by anti-GD2 CAR-M. Cancer cells were mono-cultured or co-cultured with macrophages at E:T ratio=4:1 for 20-24 hours. Statistics of cancer cell survival. Results are mean ±SD. Student’s t-test; *, $p < 0.05$; n = 3 independent experiments.

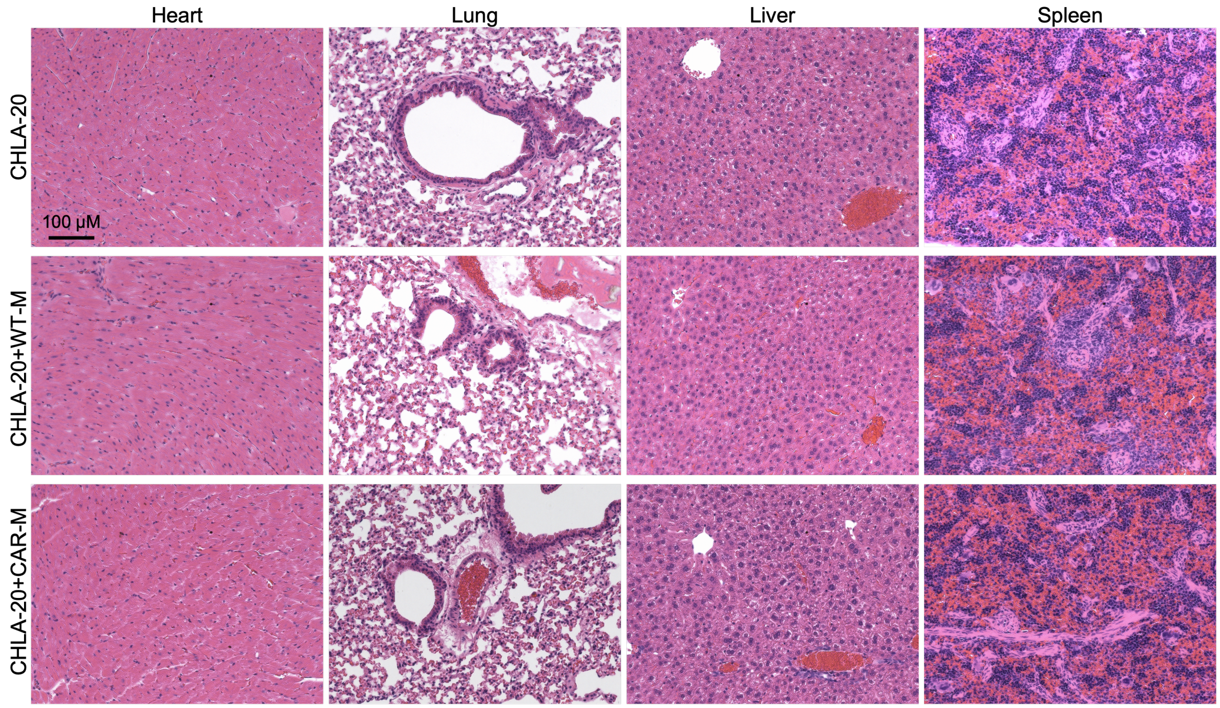

Figure S2. Histology of major organs. Related to Figure 5.

CHLA-20 cells ($1x10^6$ cells/mouse) were injected subcutaneously. Three days later, WT-M or CAR-M was injected through tail vein ($5x10^6$ cells/mouse). The mice were sacrificed 5 days later, and the organs were collected for H&E staining.

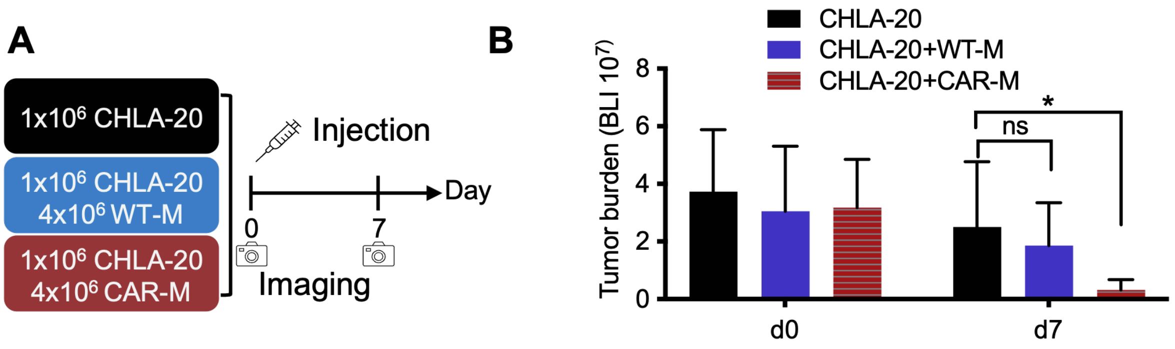

Figure S3. ***In vivo*** **antitumor activity of CAR-M derived from PBMC-3-1 hiPSCs. Related to Figure 5.**

(A) Schematic of mouse model experiments. CHLA-20-AkaLuc-GFP cells were injected alone or with WT-M or CAR-M (derived from PBMC-3-1 hiPSCs) into the hind flank of mice. Luminescent signals were measured 3 hours and 7 days post injection. 8–10 mice per group.

(B) Statistics data of tumor burden was shown by luminescent signals. Data are represented as mean ±SD. Student's t-test; *, $p < 0.05$; n = 8-10 mice.

Table S1. List of Abbreviations

CAR	Chimeric antigen receptor
CAR-M	CAR macrophage
WT-M	Wild type macrophages
PSCs	Pluripotent stem cells
NK cells	Natural killer cells
EHT	Endothelial-to-hematopoietic transition
HE	Hemogenic endothelium
FGF2	Fibroblast growth factor 2
VEGFA	Vascular growth factor A
RESV	Resveratrol
SCF	Stem cell factor
TPO	Thrombopoietin
IL-1B	Interleukin 1 beta
IL3	Interleukin 3
IL6	Interleukin 6
GM-CSF	Granulocyte-macrophage colony-stimulating factor
M-CSF	Macrophage colony-stimulating factor
IFNg	Interferon gamma
LPS	Lipopolysaccharides
GO	Gene ontology
DE	Differential expression
E:T	Effector to target
KOSR	Knockout serum replacement
FBS	Fetal bovine serum
DMSO	Dimethyl sulfoxide

Table S2 Medium components

Medium components	E8	E8BAC	E6	Five factors	FVR	SIT	M4	M5	M36	Vendor	Cat#
DF3S	+	+	+	+	+	+	+	+	+	Thermo Fisher	N/A[a]
Transferrin (10.7 μg/ml)	+	+	+	+	+	+	+	+	+	Fisher Scientific	2914-HT-001G
Insulin (20 μg/ml)	+	+	+		+	+	+	+	+	Sigma	I9287-5ML
FGF2 (100 ng/ml)	+	+		+	+					Homemade	
TGFβ1 (1.7 ng/ml)	+	+								R&D Systems	240-B
BMP4 (5 ng/ml)		+								R&D Systems	314-BP
Activin A (25 ng/ml)		+								R&D Systems	338-AC
CHIR99021 (1 μM)		+								R&D Systems	4423
VEGFA165 (50 ng/ml)				+	+					R&D Systems	293-VE
SB431542 (10 μM)				+						R&D Systems	1614
RESV (5 μM)				+	+					R&D Systems	1418
L690 (10 μM)				+						R&D Systems	0681
SCF (50 ng/ml)						+				R&D Systems	7466-SC
IL3 (10-50 ng/ml)						+			+	Peprotech	200-03
TPO (50 ng/ml)						+				Peprotech	300-18
GM-CSF(200 ng/ml)							+			Peprotech	300-03
IL-1B (10-50 ng/ml)								+		Peprotech	200-01B
M-CSF(20-100 ng/ml)								+	+	Peprotech	300-25
IL-6 (20 ng/ml)									+	Peprotech	200-06
KOSR[b] (10%)								+	+	Fisher Scientific	10828028

[a] DF3S is a customized order which contains DMEM/F12, L-ascorbic acid-2-phosphate magnesium (64 ng/ml), Sodium selenium (14 ng/ml), and $NaHCO_3$ (543 μg/ml).
[b]KOSR can be replace by FBS.

Table S3. PCR primers or probes

Primers/probes	Sequence
J507	TCGACTTCCCCTCTTCCGATG
J508	GAGCCTAGGGCCGGGATTCTC
J487	GACAGCATGTTTGCTGCCTC
J488	CCTGGTGAACACCTAGGACG
PuroR FWD Set 1	GTC ACC GAG CTG CAA GAA
PuroR REV Set 1	CCG ATC TCG GCG AAC AC
PuroR PRB Set 1	/56-FAM/TCG ACA TCG /ZEN/GCA AGG TGT GGG T/3IABkFQ/
TERT FWD Set 1	GAC CAA GCA CTT CCT CTA CTC
TERT REV Set 1	GGA ACC CAG AAA GAT GGT CTC
TERT PRB Set 1	/56-FAM/AGA GAG CTG /ZEN/AGT AGG AAG GAG GGC /3IABkFQ/
CAR FWD Set 1	GGC TTC TGG TTC CTC ATT CA
CAR REV Set 1	GGT TGT AGC TAG TTC CAC CAT AG
CAR PRB Set 1	/56-FAM/ACT GGG TGA /ZEN/GGC AGA ACA TTG GAA /3IABkFQ/
GAPDH	Assay ID: Hs02786624_g1, Thermofisher

Table S4. Antibodies

Antibody	Vendor	Cat#	Dilution
CD31-Alexa 594	Biolegend	303126	1:100
CD31-PE	BD Bioscience	555446	1:100
CD144-FITC	BD Bioscience	560411	1:100
CXCR4-APC	BD Bioscience	560936	1:100
DLL4-APC	Miltenyi	130-096-560	1:100
CD34-APC	BD Bioscience	555824	1:100
CD34-PE	BD Bioscience	560941	1:100
CD45-FITC	BD Bioscience	560976	1:100
CD14-Alexa 488	BD Bioscience	562689	1:100
CD11b-PE	Thermo Fisher	12-0118-42	1:100
Anti-GD2-CAR	Absolute Antibody	Ab02227-1.1	1:200
Anti-mouse IgG	Thermo Fisher	A31571	1:500
CD80-FITC	Fisher Scientific	305205	1:100
CD86-PE	Fisher Scientific	374205	1:100
CD163-PE/Cy7	Fisher Scientific	333613	1:100
CD206-APC	Fisher Scientific	321109	1:100
CD68-FITC	Thermo Fisher	11-0689-42	1:100
SIRPA/CD172A-PE	Biolegend	323806	1:100
GD2-FITC	Fisher Scientific	50-207-7653	1:900
CD90-PE	Biolegend	328109	1:100
CD49f-Alexa 647	BD Bioscience	561557	1:100
CD43-FITC	BD Bioscience	555475	1:100

Supplemental experimental procedures

Statistical analysis

Statistical analysis was performed with two-tailed unpaired Student's t tests as indicated in the figure legends. Data are represented as mean ±SD. A p value of <0.05 was considered to indicate statistical significance.

Maintenance of human pluripotent stem cells (hPSCs)

The experiments were performed under approval from UW–Madison Institutional Review Board. H1 and H9 embryonic stem cells and PBMC-3-1 induced pluripotent stem cells were cultured in E8 medium (Thermo Fisher customized DF3S base medium supplemented with 100 ng/ml FGF2, 1.7 ng/ml TGF-β1, 20 μg/ml insulin, and 10.7 μg/ml Transferrin) on a Matrigel-coated plate (9 μg/cm^2 or 500 μg/plate) (BD Biosciences, Cat # 354230, Batch 2104930). Refer to table 2 for more information.

CAR construct and generation of anti-GD2 CAR-PSCs.

Anti-GD2 CAR (Louis et al., 2011) was cloned into AAVS1-DEST (Oceguera-Yanez et al., 2016) vector and integrated into AAVS1 alleles using CRSPR-Cas9 as previously described (Oceguera-Yanez *et al.*, 2016). CAR integration into AAVS1 locus is confirmed by genomic PCR with the primers (J507/J508 for KI alleles and J487/J488 for WT alleles) listed in table 3.

Copy No. of AAVS1-GD2-CAR-PuroR was determined by qPCR. Genomic DNA was isolated from anti-GD2 CAR PSCs, WT PSCs, and a PCS reporter cell line with one copy

of PuroR cassette. qPCR was performed on ViiA 7 system (Life Technologies) by using the PuroR and TERT probe sets listed in table 3.

CAR sequence

ATGGAGTTTGGGCTGAGCTGGCTTTTTCTTGTGGCTATTTTAAAAGGTGTCCAGTGCTCTAGAGATAT
TTTGCTGACCCAAACTCCACTCTCCCTGCCTGTCAGTCTTGGAGATCAAGCCTCCATCTCTTGCAGAT
CTAGTCAGAGTCTTGTACACCGTAATGGAAACACCTATTTACATTGGTACCTGCAGAAGCCAGGCCA
GTCTCCAAAGCTCCTGATTCACAAAGTTTCCAACCGATTTTCTGGGGTCCCAGACAGGTTCAGTGGC
AGTGGATCAGGGACAGATTTCACACTCAAGATCAGCAGAGTGGAGGCTGAGGATCTGGGAGTTTATT
TCTGTTCTCAAAGTACACATGTTCCTCCGCTCACGTTCGGTGCTGGGACCAAGCTGGAGCTGAAACG
GGCTGATGCTGCACCAACTGTATCCATCTTCCCAGGCTCGGGCGGTGGTGGGTCGGGTGGCGAGG
TGAAGCTTCAGCAGTCTGGACCTAGCCTGGTGGAGCCTGGCGCTTCAGTGATGATATCCTGCAAGG
CTTCTGGTTCCTCATTCACTGGCTACAACATGAACTGGGTGAGGCAGAACATTGGAAAGAGCCTTGA
ATGGATTGGAGCTATTGATCCTTACTATGGTGGAACTAGCTACAACCAGAAGTTCAAGGGCAGGGCC
ACATTGACTGTAGACAAATCGTCCAGCACAGCCTACATGCACCTCAAGAGCCTGACATCTGAGGACT
CTGCAGTCTATTACTGTGTAAGCGGAATGGAGTACTGGGGTCAAGGAACCTCAGTCACCGTCTCCTC
AGCCAAAACGACACCCCCATCAGTCTATGGAAGGGTCACCGTCTCTTCAGCGGAGCCCAAATCTTGT
GACAAAACTCACACATGCCCACCGTGCCCGGATCCCAAATTTTGGGTGCTGGTGGTGGTTGGTGGA
GTCCTGGCTTGCTATAGCTTGCTAGTAACAGTGGCCTTTATTATTTTCTGGGTGAGGAGTAAGAGGA
GCAGGCTCCTGCACAGTGACTACATGAACATGACTCCCCGCCGCCCCGGGCCCACCCGCAAGCATT
ACCAGCCCTATGCCCCACCACGCGACTTCGCAGCCTATCGCTCCAGGGACCAGAGGCTGCCCCCC
GATGCCCACAAGCCCCCTGGGGGAGGCAGTTTCCGGACCCCCATCCAAGAGGAGCAGGCCGACGC
CCACTCCACCCTGGCCAAGATCAGAGTGAAGTTCAGCAGGAGCGCAGACGCCCCCGCGTACCAGC
AGGGCCAGAACCAGCTCTATAACGAGCTCAATCTAGGACGAAGAGAGGAGTACGATGTTTTGGACA
AGAGACGTGGCCGGGACCCTGAGATGGGGGGAAAGCCGAGAAGGAAGAACCCTCAGGAAGGCCT
GTACAATGAACTGCAGAAAGATAAGATGGCGGAGGCCTACAGTGAGATTGGGATGAAAGGCGAGCG
CCGGAGGGGCAAGGGGCACGATGGCCTTTACCAGGGTCTCAGTACAGCCACCAAGGACACCTACG
ACGCCCTTCACATGCAGGCCCTGCCCCCTCGCTAA

RT-qPCR of CAR expression

CAR expression was determined by RT-qPCR. Reverse transcription was performed by using SuperScript™ IV VILO™ Master Mix (Life Technologies, Cat # 11756050). qPCR was performed on ViiA 7 system (Life Technologies) by using the CAR and GAPDH probes listed in table 3.

Immunostaining of GD2-CAR

Macrophages were treated with lipopolysaccharides (LPS, 1X) (Thermo Fisher, Cat # 00-4976-03) and interferon gamma (IFNγ, 100 ng/ml) (PeproTech, Cat # 300-02) for 2–4 days to improve the cell attachment during the staining. Macrophages were fixed in cold methanol for 10 mins at -20°C and then blocked and permeabilized with 2.5% donkey serum and 0.2% Triton-X100 for 20 mins at room temperature. Anti-Disialoganglioside GD2 antibody 14G2a (Absolute Antibody, clone 1A7, Cat # Ab02227-1.1, 1:200 dilution) was added and incubated for 2 hours at room temperature. After removing the primary antibody, a secondary antibody (Thermo Fisher, Anti-mouse IgG-Alexa Flour 647, Cat # A31571, 1:500 dilution) was applied and incubated for 1 hour at room temperature. Macrophages were gently washed with PBS between each staining steps. Images were taken by Nikon confocal microscopy.

High density differentiation

The experiments were performed under approval from UW–Madison Institutional Review Board. Human PSCs were cultured in E8 medium on a Matrigel-coated plate (9 µg/cm^2 or 500 µg/plate). To achieve the best differentiation results, PSCs were split using EDTA (0.5 mM in PBS, osmolarity 340 mOsm) (Thermo Fisher, Cat # 1575020) at 1:4 ratio 2 days before differentiation. The cells reached 80–90% confluency 2 days later. At the day of differentiation (day 0), PSCs were dissociated by Accutase (Invitrogen, Cat # A1110501) for ~5 min at 37°C. To induce mesoderm differentiation, the cells were plated on a vitronectin-coated (0.9 µg/cm^2 or 50 µg/plate) (60-478 aa, homemade) plate (1.1 x 10^5 cells/cm^2) by using E8BAC medium for 2 days (usually, 44 hours). To improve cell survival, 10 µM Y27632 (R&D Systems, Cat # 1254) was used at day 0. From day 2 to

day 6, “five factor” medium was used. From day 6 to day 10, FVR medium was used. Please refer to table 2 for more information about the media.

Low density differentiation optimized for generation of hematopoietic progenitors

To induce mesoderm differentiation, PSCs were plated on a vitronectin-coated (0.9 μg/cm^2, or 50 μg/plate) plate (1.8 x 10^4 cells/cm^2) by using E8BAC media for 2 days (usually, 44 hours). To improve cell survival, 10 μM Y27632 was used at day 0. From day 2 to day 6, “five factor” medium was used. From day 6 to day 10, FVR medium was used. The day 10 cells can be used for macrophage differentiation or further expanded in SIT medium for 3 days in order to enhance proportion and yield of $CD34^+CD45^+$ progenitors.

From day 0–6: medium (12ml/ plate) was changed every day.

From day 6–10: medium (24 ml/ plate) was changed every other day.

Please refer to table 2 for more information about the media.

The day 10 or day 13 hematopoietic progenitor cells can be cryopreserved in 90% fetal bovine serum (FBS) with 10% dimethyl sulfoxide (DMSO).

An alternative protocol for generation of hematopoietic progenitors

An alternative protocol can be used to further improve hematopoietic progenitor generation. The PSCs were plated on a vitronectin-coated plate at high density (1.1 x 10^5 cells/cm^2) in E8BAC medium for 2 days. The mesoderm cells were passaged and seeded on a new vitronectin-coated plate at low density (about 1:8 split, 0.7-1.1 x 10^5 cells/cm^2). From day 2 to day 6, “five factor” medium was used. From day 6 to day 10, FVR medium was used.

The mesoderm cells can be cryopreserved in E8BAC medium with 10% DMSO, so it saves the time for PSCs culture and mesoderm differentiation.

The hematopoietic progenitor cells can be cryopreserved in 90% fetal bovine serum (FBS) with 10% dimethyl sulfoxide (DMSO).

Please refer to table 2 for more information about the media.

Macrophage differentiation

The round shape hematopoietic progenitor cells were collected from above hematopoietic differentiation protocol on day 10, transferred to low-adhesion plate, and cultured at a concentration of 1 x 10^6 cells/ml in M4 media for 3 days. Three days later, the cells were transferred to 10-cm dish or 6-well plate (non-coating, regular cell culture plate) (1-2 x 10^4 cells/cm^2) with M5 media for another 7–15 days. Half of the M5 was changed every 3–4 days.

A modified protocol can be used to further improve the CAR-M differentiation. The PSCs were plated on a vitronectin-coated plate (1.1 x 10^5 cells/cm^2) in E8BAC medium for 2 days. The mesoderm cells were passaged and seeded on a new vitronectin-coated plate (0.7-1.1 x 10^5 cells/cm^2). From day 2 to day 7, “five factor” media was used. From day 7 to day 11, FVR media was used. The day 11 hematopoietic progenitor cells were transferred to a new plate (non-coating, regular cell culture plate) and cultured in M36 media for 3 days (day 11–14, 1 x 10^6 cells/ml). The cells were them transferred to another new plate (non-coating, regular cell culture plate) (1-2 x 10^4 cells/cm^2) with M5 media for another 7–15 days. Half of the M5 was changed every 4 days.

Please refer to table 2 for more information about the media.

Flow cytometry

Cells (0.1-1 x 10^6) were resuspended in diluted antibodies and incubated at 4°C for 0.5–1 hour. Cells were then washed with 2% FBS-PBS for 1–2 times and cytometric analysis was performed on BD FACSCanto II. FlowJo was used for the data analysis.

Please refer to table 4 for more information about the antibody.

RUNX1-venus reporter cell line

RUNX1+23-Venus reporter cell line was previously generated by Slukvin lab (Uenishi et al., 2018).

Hematopoietic colony forming unit assay

Hematopoietic colony forming unit assay was conducted in serum-containing Methocult (Stem Cell Technologies, Cat # H4436) according to manufacturer instruction. Day 8 cells (both the attached and floating cells) were collected for analysis.

Phagocytosis of BioParticles

Macrophages were seeded on 12-well plate (2 x 10^5 cells/well, non-coating) with M5 medium. Add 100 μl of 1mg/ml Zymosan A BioParticles (2 x 10^7 particles/mg solid) (Thermo Fisher, Cat # Z2843) to each well. Phagocytosis was imaged over a 24 hour time period (image capture every 10–30 minutes) (Nikon, BioCT).

***In vitro* cytotoxicity/phagocytosis assay**

AkaLuc-GFP was cloned into AAVS1-DEST vector and then integrated into AAVS1 locus of CHLA-20, WM266-4, K562, and Raji cells by using CRISPR-Cas9 technology (Oceguera-Yanez *et al.*, 2016). Arterial endothelial cells and smooth muscle cells were derived from NOS3-NanoLuc-tdTomato and MYH11-NanoLuc-tdTomato H1 cell lines, respectively, using previously described protocols ((Zhang et al., 2017; Zhang et al., 2019)). To assess cytotoxicity, cells were incubated at indicated effectors:targets (E:T) ratio of 3:1 for 20–24 hours in 96-well plate. Luciferase substrate (50–250 µM Tokeoni for AkaLuc, R&D Systems, Cat # 6555) (Nano-Glo for NanoLuc, Promega, Cat # N1120, 1:1000 dilution) was added to the cell culture and bioluminescence was measured using Promega GloMax plate reader 15 minutes later.

For imaging, macrophages were seeded in 96-well plate (2-6 x 10^4 cells/well, non-coating) with M5 media with or without CHLA-20 (2 x 10^4 cells/well). Images were taken on EVOS Cell Imaging System (Thermo Fisher). Time-lapse imaging was taken over a 20–24 hour time period (image capture every 20 min) by using BioCT (Nikon).

In vivo anti-tumor activity analysis

The experiments were performed under approval from UW–Madison, Institutional Review Board. NOD-scid IL2Rgamma^{null} (NSG) mice (6 to 8 week old) were obtained from Jackson Lab. Neuroblastoma cells CHLA20-AkaLuc-GFP and macrophages were injected into the hind flank of the mice. Antitumor effect was monitored by bioluminescent imaging using IVIS imaging system at the indicated time (100 µl 5mM Tokeoni/mouse).

Secretome assay

Secretome assay was performed according to the manufacturer's instruction (Mesoscale, U-PLEX Macrophage M1 Combo 1, Cat # K15336K).

Statistical analysis

P value was calculated by Student's t-test, tails = 2, type = 2.

RNA-sequencing

WT-M and CAR-M were co-cultured with CHLA-20 cells (E:T = 3:1) for 1 day as described in "*in vitro* cytotoxicity assay" and then $CD14^{+}CD11b^{+}$ macrophage were sorted by flowcytometry (BD FACSAria). Total RNA was isolated from the co-cultured purified macrophages or mono-cultured macrophages by using the RNeasy Plus Micro Kit (Qiagen, Cat # 74034) and quantified with both the Quant-It RNA Assay Kit (Thermo Fisher, Q10210) and the Bioanalyzer RNA 6000 Pico Kit (Agilent). The cDNA library was prepared using a custom LM-Seq protocol previously described in the previous study(Hou et al., 2015). The final indexed cDNA libraries were pooled with 19 samples per lane and run on the NextSeq 2000 (Illumina).

Read Processing

The sequencer outputs were processed using Illumina's Dragen 3.8.4 BCLConvert software. Adapter sequences and low-quality ends were trimmed from reads. Each sample's reads were then processed using RSEM version 1.2.3 (with bowtie-0.12.9 for the alignment step), aligning to a HG38 reference. RSEM input parameters were specified such that alignments were suppressed for a read if over 200 valid alignments existed ('--

bowtie-m' option), a maximum of two mismatches were allowed in the seed ('-- bowtie-n' option), and the probability of generating a read from the forward strand of the transcript was 0.5 since reads were not strand-specific ('--forwardprob' option).

Differential Expression Analysis

Differentially Expressed genes were determined using DESeq2 v1.28.1, running under R v4.0.

Gene Ontology (GO) Analysis and Heatmaps

The most highly differentially expressed genes were then subjected to GO analysis. A threshold of unadjusted p-val <= 0.05 was used, resulting in 807 and 868 down-regulated and up-regulated genes respectively. Gene set enrichment analysis on the differential expression (DE) genes was performed using GOSeq (Young et al., 2010) using all Biological Process gene sets in the Gene Ontology downloaded from MSigDB (Subramanian et al., 2005). GO ontology terms related to macrophages were chosen for display. For heatmapping, the gene expression data was normalized by computing log(TPM+1) and then for each gene, the Z-score was computed by subtracting the mean and dividing by the standard deviation over all of the samples selected for the heatmap.

References

Hou, Z., Jiang, P., Swanson, S.A., Elwell, A.L., Nguyen, B.K., Bolin, J.M., Stewart, R., and Thomson, J.A. (2015). A cost-effective RNA sequencing protocol for large-scale gene expression studies. Sci Rep *5*, 9570. 10.1038/srep09570.

Louis, C.U., Savoldo, B., Dotti, G., Pule, M., Yvon, E., Myers, G.D., Rossig, C., Russell, H.V., Diouf, O., Liu, E., et al. (2011). Antitumor activity and long-term fate of chimeric antigen receptor-positive T cells in patients with neuroblastoma. Blood *118*, 6050-6056. 10.1182/blood-2011-05-354449.

Oceguera-Yanez, F., Kim, S.I., Matsumoto, T., Tan, G.W., Xiang, L., Hatani, T., Kondo, T., Ikeya, M., Yoshida, Y., Inoue, H., and Woltjen, K. (2016). Engineering the AAVS1 locus for consistent and scalable transgene expression in human iPSCs and their differentiated derivatives. Methods *101*, 43-55. 10.1016/j.ymeth.2015.12.012.

Subramanian, A., Tamayo, P., Mootha, V.K., Mukherjee, S., Ebert, B.L., Gillette, M.A., Paulovich, A., Pomeroy, S.L., Golub, T.R., Lander, E.S., and Mesirov, J.P. (2005). Gene set enrichment analysis: a knowledge-based approach for interpreting genome-wide expression profiles. Proc Natl Acad Sci U S A *102*, 15545-15550. 10.1073/pnas.0506580102.

Uenishi, G.I., Jung, H.S., Kumar, A., Park, M.A., Hadland, B.K., McLeod, E., Raymond, M., Moskvin, O., Zimmerman, C.E., Theisen, D.J., et al. (2018). NOTCH signaling specifies arterial-type definitive hemogenic endothelium from human pluripotent stem cells. Nat Commun *9*, 1828. 10.1038/s41467-018-04134-7.

Young, M.D., Wakefield, M.J., Smyth, G.K., and Oshlack, A. (2010). Gene ontology analysis for RNA-seq: accounting for selection bias. Genome Biol *11*, R14. 10.1186/gb-2010-11-2-r14.

Zhang, J., Chu, L.F., Hou, Z., Schwartz, M.P., Hacker, T., Vickerman, V., Swanson, S., Leng, N., Nguyen, B.K., Elwell, A., et al. (2017). Functional characterization of human pluripotent stem cell-derived arterial endothelial cells. Proc Natl Acad Sci U S A *114*, E6072-E6078. 10.1073/pnas.1702295114.

Zhang, J., McIntosh, B.E., Wang, B., Brown, M.E., Probasco, M.D., Webster, S., Duffin, B., Zhou, Y., Guo, L.W., Burlingham, W.J., et al. (2019). A Human Pluripotent Stem Cell-Based Screen for Smooth Muscle Cell Differentiation and Maturation Identifies Inhibitors of Intimal Hyperplasia. Stem Cell Reports *12*, 1269-1281. 10.1016/j.stemcr.2019.04.013.
