# Supplementary material for: Generation of anti-GD2 CAR macrophages from human pluripotent stem cells for cancer immunotherapies
Source: Stem Cell Reports. 2023 Jan 12;18(2):585–96. doi: 10.1016/j.stemcr.2022.12.012 (PMC9968983; doi:10.1016/j.stemcr.2022.12.012)
Supplement: Document S2. Article plus supplemental information [file mmc5.pdf]

Stem Cell Reports
Article
OPEN ACCESS

Generation of anti-GD2 CAR macrophages from human pluripotent stem cells for cancer immunotherapies

Jue Zhang,[1] Sarah Webster,[1] Bret Duffin,[1] Matthew N. Bernstein,[1] John Steill,[1] Scott Swanson,[1] Matthew H. Forsberg,[2] Jennifer Bolin,[1] Matthew E. Brown,[3] Aditi Majumder,[4] Christian M. Capitini,[2,5] Ron Stewart,[1] James A. Thomson,[1,8,*] and Igor I. Slukvin[4,6,7,8,*]

[1]Morgridge Institute for Research, Madison, WI 53715, USA
[2]Department of Pediatrics, University of Wisconsin–Madison, Madison, WI 53792, USA
[3]Department of Surgery, University of Wisconsin–Madison, Madison, WI 53792, USA
[4]Wisconsin National Primate Research Center, University of Wisconsin–Madison, Madison, WI 53715, USA
[5]Carbone Cancer Center, University of Wisconsin–Madison, Madison 53705, WI, USA
[6]Department of Cell & Regenerative Biology, University of Wisconsin–Madison, Madison, WI 53706, USA
[7]Department of Pathology and Laboratory Medicine, University of Wisconsin–Madison, Madison, WI 53705, USA
[8]Senior author
*Correspondence: jthomson@morgridge.org (J.A.T.), islukvin@wisc.edu (I.I.S.)
https://doi.org/10.1016/j.stemcr.2022.12.012

SUMMARY

Macrophages armed with chimeric antigen receptors (CARs) provide a potent new option for treating solid tumors. However, genetic engineering and scalable production of somatic macrophages remains significant challenges. Here, we used CRISPR-Cas9 gene editing methods to integrate an anti-GD2 CAR into the AAVS1 locus of human pluripotent stem cells (hPSCs). We then established a serum- and feeder-free differentiation protocol for generating CAR macrophages (CAR-Ms) through arterial endothelial-to-hematopoietic transition (EHT). CAR-M produced by this method displayed a potent cytotoxic activity against GD2-expressing neuroblastoma and melanoma *in vitro* and neuroblastoma *in vivo*. This study provides a new platform for the efficient generation of off-the-shelf CAR-Ms for antitumor immunotherapy.

INTRODUCTION

Chimeric antigen receptor (CAR)-T cell and CAR-natural killer (NK) cell therapies have already demonstrated tremendous success in the eradication of lymphoid malignancies (Alcantara et al., 2018; Liu et al., 2020). However, many challenges remain in the application of CAR therapies for solid tumors (Klichinsky et al., 2020; Marofi et al., 2021a, 2021b), and responses with CAR-T cells have been limited to isolated, exceptional cases (Ahmed et al., 2015, 2017; Brown et al., 2016; Heczey et al., 2020; Hegde et al., 2017; Louis et al., 2011; Pule et al., 2008; Wang et al., 2018). Given the known capacity of macrophages to infiltrate and reside within solid tumors, CAR macrophages (CAR-Ms) have been proposed as a novel tool for immunotherapy of these tumors (Guedan et al., 2019; Klichinsky et al., 2020; Morrissey et al., 2018; Zhang et al., 2020). Despite that, generating macrophages from somatic cells represents a significant challenge, and transduction of monocytes/macrophages with lentiviruses is inefficient. While monocytes/macrophages can be transduced efficiently with adenoviral vectors, these vectors do not integrate into genomes and may not sustain prolonged CAR expression. Human pluripotent stem cells (hPSCs) are a logical alternative for large-scale production of CAR-Ms of uniform quality for off-the-shelf immunotherapy.

As a CAR target antigen, the disialoganglioside GD2 has been widely studied due to its high expression on solid tumors like neuroblastoma and melanoma, as well as documented improvement in survival with US Food and Drug Administration-approved anti-GD2 therapies like dinutuximab and naxitamab (Cheung et al., 2021; Yu et al., 2010). Since neuroblastoma is the most common and difficult-to-treat pediatric solid tumor, and melanoma remains the most lethal of the primary cutaneous neoplasms (Matthay et al., 2016; Switzer et al., 2022), we aim to generate hPSCs with an anti-GD2 CARs integrated into the AAVS1 locus and then differentiate them into macrophages by applying our arterial endothelium differentiation platform.

Using a *EFNB2-tdTomato/EPHB4-EGFP* dual-reporter hPSC line, we previously identified factors that regulate arterial endothelial cell specification and developed a method for the efficient generation of arterial endothelium in xeno-free, fully defined conditions (Zhang et al., 2017). In this study, we demonstrated that reducing cell-cell contact via low-density cell seeding produced arterialized endothelial cells with the capacity to undergo endothelial-to-hematopoietic transition (EHT), leading to the formation of blood cells. As we previously reported, arterial hemogenic endothelium (HE) is enriched in definitive lympho-myeloid progenitors, while nonarterial HE possesses mostly myeloid potential (Jung et al., 2021; Uenishi et al., 2018). In the present study, we created a new hPSC-derived arterial HE platform to generate macrophages expressing anti-GD2 CAR, which displayed potent antitumor activity against neuroblastoma and melanoma *in vitro* and neuroblastoma *in vivo*.

This is an open access article under the CC BY license (http://creativecommons.org/licenses/by/4.0/).

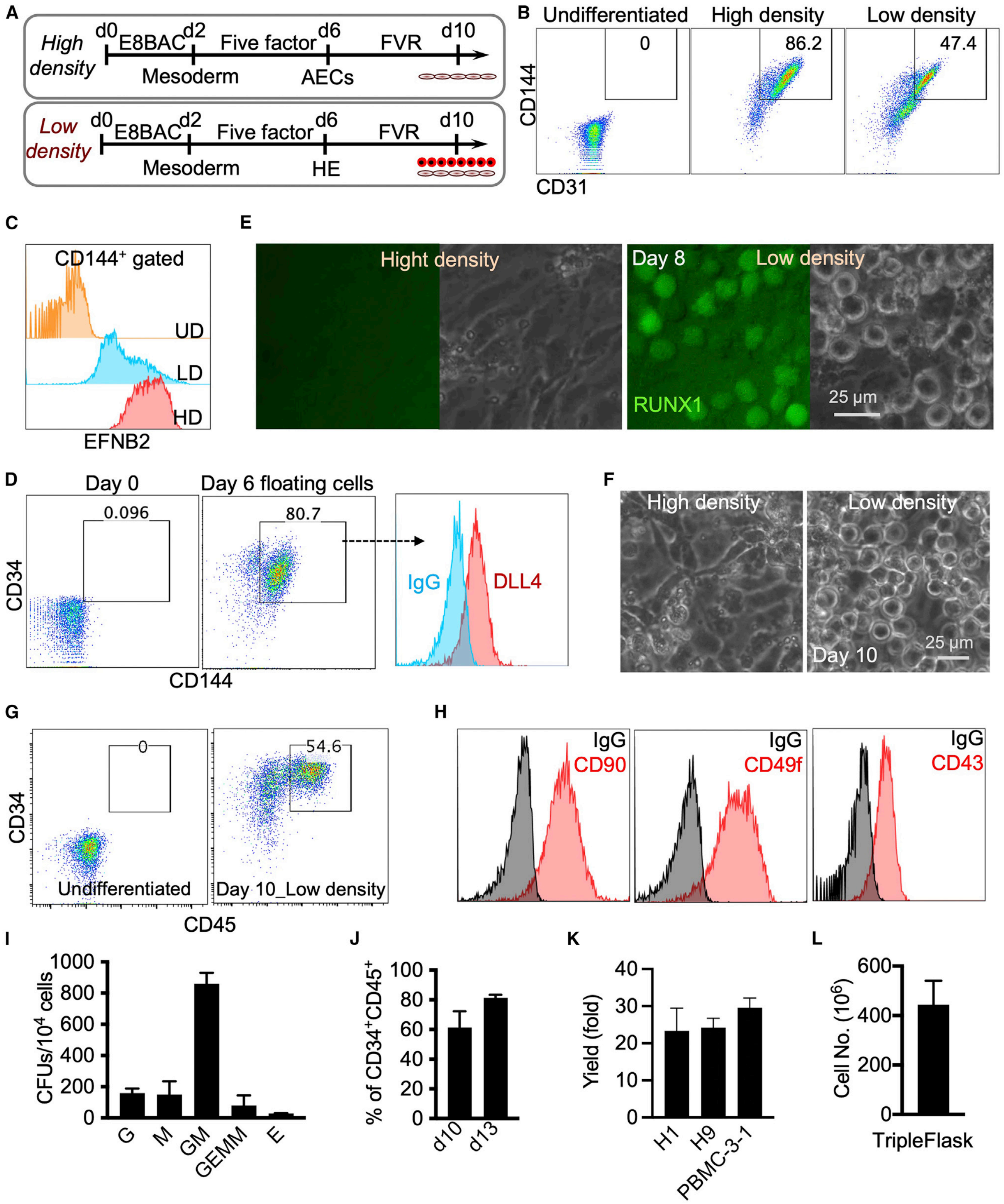

(legend on next page)

RESULTS

Generation of hematopoietic progenitors using a modified arterial endothelium differentiation protocol

We have previously established a highly efficient, fully defined, xeno-free protocol for differentiation of arterial endothelial cells from hPSCs (Zhang et al., 2017). However, this protocol did not lead to blood formation where arterial endothelial cell differentiation was performed with hPSCs seeded at high cell density (Figure 1A) (Zhang et al., 2017). Since the strengthening of tight junctions between aortic endothelial cells in high-density cultures may repress specification of HE and subsequent EHT (Zhang et al., 2014), we hypothesized that reducing cell-cell contact by seeding the cells at low density may allow for the generation of arterial endothelium with hemogenic potential. To test this hypothesis, we used differentiation conditions and media similar to our prior protocol but decreased the initial cell density to 1.8×10^4 versus 1.1×10^5 cells/cm^2 density in our original protocol (Figure 1A). Decreasing the cell density reduced the proportion of CD31$^+$CD144$^+$ endothelial cells in day 6 differentiation cultures (Figure 1B). However, these endothelial cells still retained arterial fate as evidenced by EFNB2 expression (Figure 1C). Additionally, a few rounds, floating cells were observed on day 6 of differentiation in the low-density protocol. These cells co-expressed CD144 and CD34, as well as moderate levels of DLL4 (Figure 1D), consistent with their origin from arterial HE.

To further induce hematopoiesis, the medium was then switched to fibroblast growth factor 2 (FGF2), vascular growth factor A (VEGFA), and resveratrol (RESV) containing medium (FVR medium), and cells were cultured for 4 more days (Figure 1A). Using the RUNX1+23-Venus enhancer-reporter H1 hPSC line (Uenishi et al., 2018), we observed that round, nonadherent, RUNX1+23$^+$ blood cells formed at day 8 of differentiation in low-density, but not in high-density, conditions (Figure 1E). At day 10, more floating hematopoietic cells expressing CD34 and CD45 were observed in the cultures (Figures 1F and 1G). These hematopoietic cells also expressed CD90, CD49f, and CD43 (Figure 1H) and displayed multilineage differentiation potential via a colony-forming unit (CFU) assay (Figure 1I). The numbers and percentages of CD34$^+$ CD45$^+$ cells could be further increased up to 80% purity after culture for an additional 3 days (Figure 1J). This protocol produced more than 20 hematopoietic progenitors from one starting hPSC from various pluripotent cell lines (H1, H9, and PBMC-3-1) and routinely generated 4×10^8 hematopoietic progenitors from a single T500 TripleFlask (Figures 1K and 1L). Together, these data demonstrate that reducing cell-cell contact by seeding cells at low density supports formation of arterial HE with the capacity to undergo EHT and formation of CD34$^+$ hematopoietic progenitors expressing DLL4.

Generation and characterization of CAR-Ms

To generate macrophages using our optimized arterial endothelium differentiation protocol, we collected the hematopoietic cells on day 10 differentiation and cultured them in serum-free media containing granulocyte-macrophage colony-stimulating factor (GM-CSF) for 3 days and then interleukin-1β (IL-1β) and macrophage CSF (M-CSF)

Figure 1. Low-density arterial endothelium differentiation cultures allow for the generation of blood cells
H9 cells are used unless specified.
(A) Schematic of arterial endothelial cell and hematopoietic cell differentiation. High cell density, 1.1×10^5 cells/cm^2. Low cell density, 1.8×10^4 cells/cm^2. FVR media contains FGF2, VEGFA, and RESV.
(B) Representative flow cytometry dot plots show expression of CD31 and CD144 at day 6 of differentiation in low- and high-density conditions. Undifferentiated cells (day 0) are used as a negative control.
(C) CD144$^+$ cells in low density (LD) and high density (HD) were gated for analysis of EFNB2 expression. Undifferentiated cells (UDs) are used as a negative control. The *EFNB2-tdTomato/EPHB4-EGFP* H1 reporter cell line was used.
(D) Representative flow cytometry analysis of CD144, CD34, and DLL4 expression of floating cells collected at day 6 of differentiation.
(E) RUNX1+23 enhancer activity in LD and HD cultures at day 8 of differentiation. The RUNX1+23 enhancer-Venus reporter cell line was used.
(F) Phase contrast images of arterial endothelium cultures at day 10 of differentiation.
(G) Representative flow cytometry analysis of CD34 and CD45 expression at day 10 of differentiation.
(H) Representative flow cytometry analysis of CD90, CD49f, and CD43 expression of floating cells collected at day 10 of differentiation.
(I) Colony-forming unit assay of day 8 cells in low cell density condition. Data are represented as mean ± SD; n = 3 independent experiments.
(J) Percentages of CD34$^+$CD45$^+$ cells at days 10 and 13 of differentiation. Data are represented as mean ± SD. Student's t test; *$p < 0.05$; n = 3 independent experiments.
(K) Hematopoietic cells generated from one starting hPSC. H1 and H9 embryonic stem cells and PBMC-3-1 hiPSCs were used. Data are represented as mean ± SD; n = 3 independent experiments.
(L) Total floating hematopoietic cell number generated from one T500 TrypleFlask. Data are represented as mean ± SD; n = 3 independent experiments.

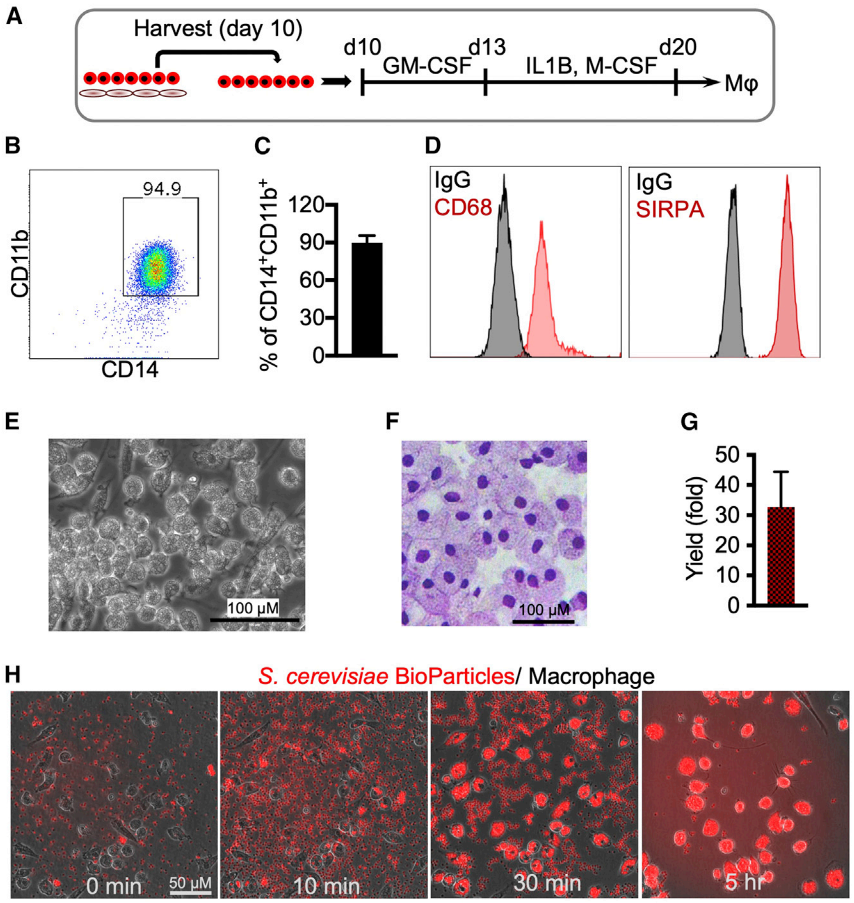

Figure 2. Generation and functional assessment of macrophages generated from H9 hPSCs

(A) Schematic of macrophage differentiation. Hematopoietic cells on day 10 of differentiation are collected and cultured in serum-free media containing GM-CSF for 3 days and then with IL-1β and M-CSF for another 7 days.

(B) Representative dot plot shows flow cytometry analysis of CD14 and CD11b expression at day 20 of differentiation.

(C) Percentages of $CD14^{+}CD11b^{+}$ macrophages are presented as mean ± SD, n = 3 independent experiments.

(D) Representative histograms show flow cytometry analysis of CD68 and SIRPA/CD172A expression at day 20 of differentiation.

(E) Cell morphology at day 20 of differentiation.

(F) Wright-Giemsa staining of cytospins from day 20 differentiation.

(G) Total yield of macrophages from one starting hPSC. Data are represented as mean ± SD, n = 3 independent experiments.

(H) Phagocytosis of yeast particles by macrophages. Zymosan An *S. cerevisiae* BioParticles (Texas Red conjugate; Life Technologies) were prepared in phosphate-buffered saline (PBS; 10 mg/mL = 2×10^{9} particle/mL). 20 μL particles were added to 2 mL media containing 4×10^{5} macrophage. Phagocytosis was imaged over time.

for another 7 days (Figure 2A). The protocol generated ~90% of $CD14^{+}CD11b^{+}$ macrophages, which also expressed CD68 and SIRPA/CD172A (Figures 2B–2D). In addition, they were large in size (20 μm diameter) and possessed typical macrophage morphology (Figures 2E and 2F). The final yield of macrophages was approximately 30-fold from the starting hPSCs (Figure 2G).

To characterize the phagocytotic function of the macrophages, BioParticles were added to macrophage cultures. The results revealed that these macrophages were functional and able to uptake yeast particles (Figure 2H; Video S1).

To generate macrophages with antigen-dependent antitumor potential, we applied CRISPR-Cas9 technology to knock in a third-generation anti-GD2 CAR (14G2a-CD28-OX40-CD3ζ) into the AAVS1 locus of H9 hPSCs (Figures 3A–3D). GD2-CAR hPSCs were then differentiated into anti-GD2 CAR-Ms (Figure 3E), which displayed similar morphology with wild-type macrophages (WT-Ms) (Figure 3F). qRT-PCR and immunostaining confirmed the expression of anti-GD2 CAR in CAR-Ms (Figures 3G and 3H). Macrophages can be polarized into M1 (antitumor, pro-inflammatory) and M2 (protumor, antiinflammatory) macrophages (Pan et al., 2020). To characterize the M1/M2 phenotypes, we treated macrophages with lipopolysaccharide (LPS) and interferon γ (IFNγ) to induce M1 polarization or IL-4 to induce M2 polarization. The results revealed that CD80 and CD86 (M1 markers) expression was greatly increased by LPS and IFNγ in both WT-Ms and CAR-Ms (Figures 3I and 3J). In addition, LPS and IFNγ decreased CD163 expression (an M2 marker) in CAR-Ms but not WT-Ms (Figures 3I and 3J). On the other hand, IL-4 increased CD206 expression (an M2 marker) in both WT-Ms and CAR-Ms (Figures 3I and 3J).

To further characterize the CAR-Ms, we performed bulk RNA sequencing of WT-Ms and CAR-Ms collected from differentiation cultures and from co-cultures with GD2-expressing CHLA-20 neuroblastoma. Gene Ontology (GO) analysis of differentially expressed genes in WT-Ms and CAR-Ms before co-culture with tumor cells revealed that only two M1-related pathways, antigen processing and presentation of peptide antigen and phagocytosis, were enriched in CAR-Ms when compared with WT-Ms (Figure 3K). After co-culture with CHLA-20 tumor cells, more M1-related

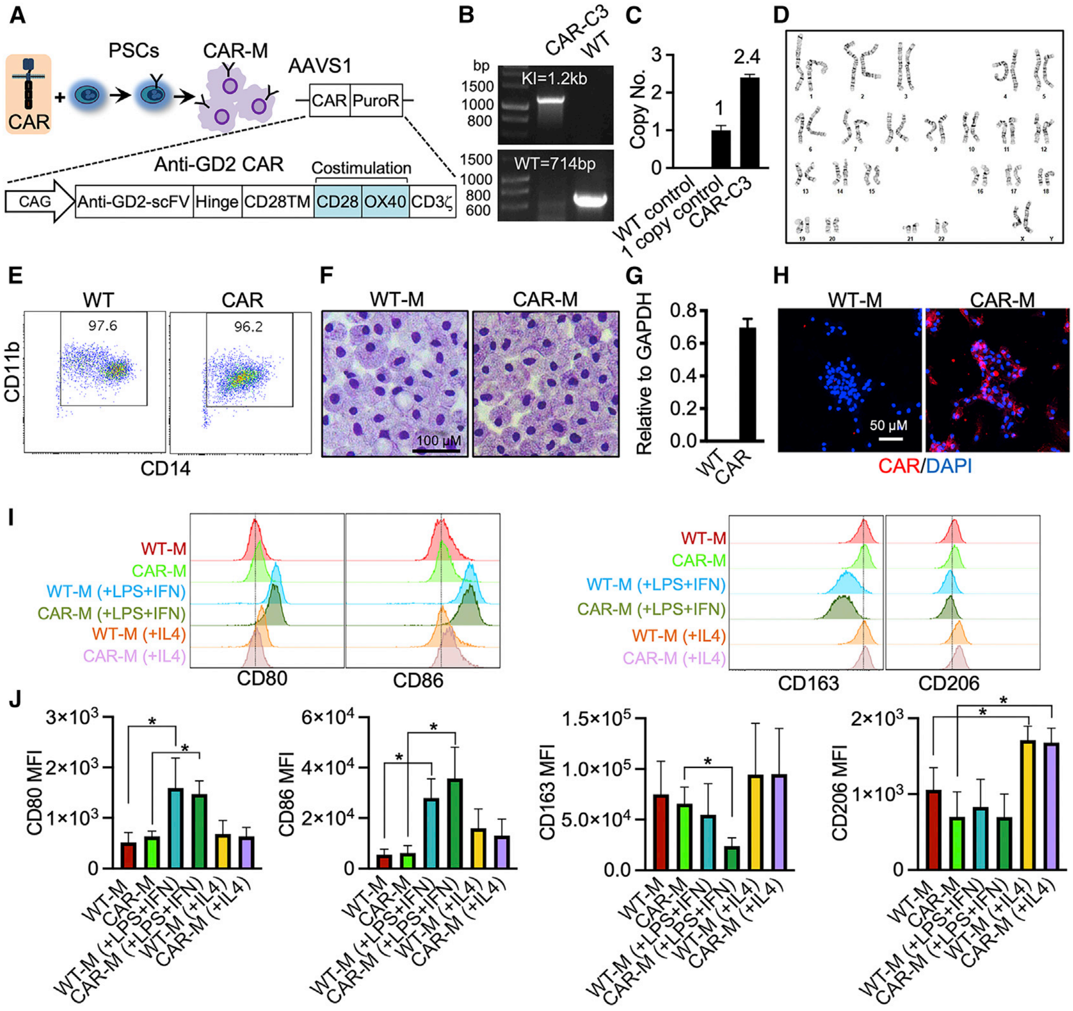

K

WT-M vs CAR-M

Category	FDR
Antigen processing and presentation of peptide antigen	4.95E-11
Phagocytosis	0.041826

L

WT-M +CHLA-20 vs CAR-M +CHLA-20

Category	FDR
Response to type I interferon	1.38E-06
Inflammatory response	0.000192
Response to interferon gamma	0.000251
Cellular response to reactive oxygen species	0.002838
Leukocyte migration involved in inflammatory response	0.007687
Leukocyte chemotaxis involved in inflammatory response	0.019192

M

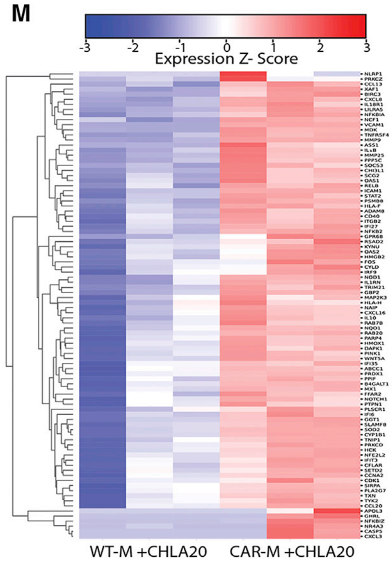

(legend on next page)

pathways were enriched in CAR-Ms, namely response to type I IFN, inflammatory response, response to IFNγ, cellular response to reactive oxygen species, leukocyte migration involved in inflammatory response, and leukocyte chemotaxis involved in inflammatory response (Figure 3L). Heatmap analysis also revealed the upregulation of numerous M1-related genes in CAR-Ms co-cultured with CHLA-20 (Figure 3M). These results suggest that CAR expression in hPSC-derived macrophages promotes their polarization toward an M1 state after co-culture with tumor cells.

Antitumor activity of CAR-Ms

Next, we evaluated the antitumor activity of CAR-Ms *in vitro*. WT-Ms or CAR-Ms were co-cultured with CHLA-20-AkaLuc-GFP neuroblastoma cells or WM266-4-AkaLuc-GFP melanoma cells at different effector-to-target (E:T) ratios. CAR-Ms demonstrated significant antitumor activity at a 1:1 E:T ratio versus CHLA-20 cells using a luciferase-based cytotoxicity assay (Figure 4A). Antitumor activity was further improved with the increased E:T ratio (Figure 4A). CAR-Ms were also able to inhibit WM266-4 cell growth at a 3:1 or higher E:T ratio (Figure 4B). In contrast, WT-Ms showed no antitumor activity against neuroblastoma and melanoma cells and appeared to promote tumor growth (Figures 4A and 4B). Time-lapse results also demonstrated that CAR-Ms were able to eliminate CHLA-20-AkaLuc-GFP cells in 1 day (Videos S2 and S3).

To further explore the interaction between tumor cells (GFP labeled) and macrophages (SIRPA stained), we performed flow cytometric analysis of co-cultured cells at different time points (Figure 4C). At 24 h, CHLA-20 cells were not reduced by WT-Ms, while most of the CHLA-20 cells were eliminated by CAR-Ms (Figure 4C). The GFP^+ $SIRPA^+$ cells were found at 1 h of CHLA-20 and CAR-M co-culture by flow cytometry (Figure 4C), indicating that CHLA-20 cells were engulfed by CAR-Ms. Fluorescence imaging further confirmed phagocytosis of CHLA-20 cells by CAR-Ms (Figure 4D, yellow arrows indicated).

To investigate whether the antitumor activity of CAR-Ms was dependent on the M1/M2 phenotype, we pre-treated WT-Ms and CAR-Ms with LPS and IFNγ or IL-4. Interestingly, although LPS and IFNγ were able to enhance M1 marker expression (Figures 3I and 3J), they failed to improve the antitumor activity of both WT-Ms and CAR-Ms (Figure 4E). Instead, the antitumor activity of CAR-Ms was slightly reduced (Figure 4E). IL-4 promoted M2 polarization (Figures 3I and 3J) but did not suppress the antitumor activity of CAR-Ms (Figure 4E). The results suggested that the antitumor activity of CAR-Ms was independent of the M1/M2 phenotype induced by LPS, IFNγ, and IL-4.

To assess the antitumor specificity, we co-cultured CAR-Ms with $GD2^-$ allogeneic healthy cells: arterial endothelial cells (AEC-NOS3-NanoLuc-2A-tdTomato) and smooth muscle cells (SMC-MYH11-NanoLuc-2A-tdTomato), as well as $GD2^-$ tumor cells: Raji-AkaLuc-GFP and K562-AkaLuc-GFP (Figures 4F and 4G). Luminescence results revealed that CAR-Ms had minimal activity against these cells (Figure 4G), demonstrating the antigen specificity of anti-$GD2^+$ tumor activity. CAR-T cell therapy could trigger life-threatening cytokine-release syndrome (CRS), so we measured the cytokine release of macrophages before and after they were co-cultured with CHLA-20 cells. CRS-related cytokines can increase 30- to 8,000-fold upon CAR-T injection (Lee et al., 2019; Norelli et al., 2018). In our results, IL-6, IP-10, MIP-1α, and tumor necrosis factor α (TNF-α) were only increased 2- to 4-fold in CAR-Ms after co-culture with CHLA-20 (Figure 4H), indicating minimal risk of CRS from CAR-Ms.

To demonstrate that our CAR-M protocol is applicable to other hPSC lines, we generated another anit-GD2

Figure 3. Engineering anti-GD2 CAR hPSCs and functional analysis of anti-GD2 CAR-Ms (H9 derived)

(A) Schematic of CAR constructs and CAR-engineered cells. CAG, CMV enhancer/chicken β action promoter; scFv, single chain fragment variable; TM, transmembrane.

(B) Junctional PCR analysis AAVS1-CAR knockin (KI) allele and WT AAVS1 allele to demonstrate a correct CAR integration. WT cells (without gene editing) are used as a control.

(C) qPCR analysis of AAVS1-GD2-CAR-PuroR copy number. Data are represented as mean ± SD. n = 2 independent experiments.

(D) Karyotyping of CAR-C3 hPSC line.

(E) Flow cytometry analysis of CD14 and CD11b expression of macrophages.

(F) Wright-Giemsa staining of WT-M and CAR-M cytospins.

(G) qRT-PCR analysis of CAR expression in macrophages. n = 3 independent experiments.

(H) Demonstration of anti-GD2 CAR expression in macrophages using immunofluorescence staining with antibody against GD2 antibody 14G2a.

(I) Representative flow cytometry plots show expression of M1/M2 markers in WT-Ms and CAR-Ms with or without treatment.

(J) Statistics of M1/M2 markers. MFI, mean fluorescent intensity. Data are presented as mean ± SD. Student's t test; $^*p < 0.05$; n = 4 independent experiments. CAR-Ms are generated from 2 different clones.

(K) M1-related GO terms enriched in CAR-Ms.

(L) M1-related GO terms enriched in CAR-Ms co-cultured with CHLA-20 cells.

(M) Heatmap shows M1-related genes in macrophages co-cultured with CHLA-20.

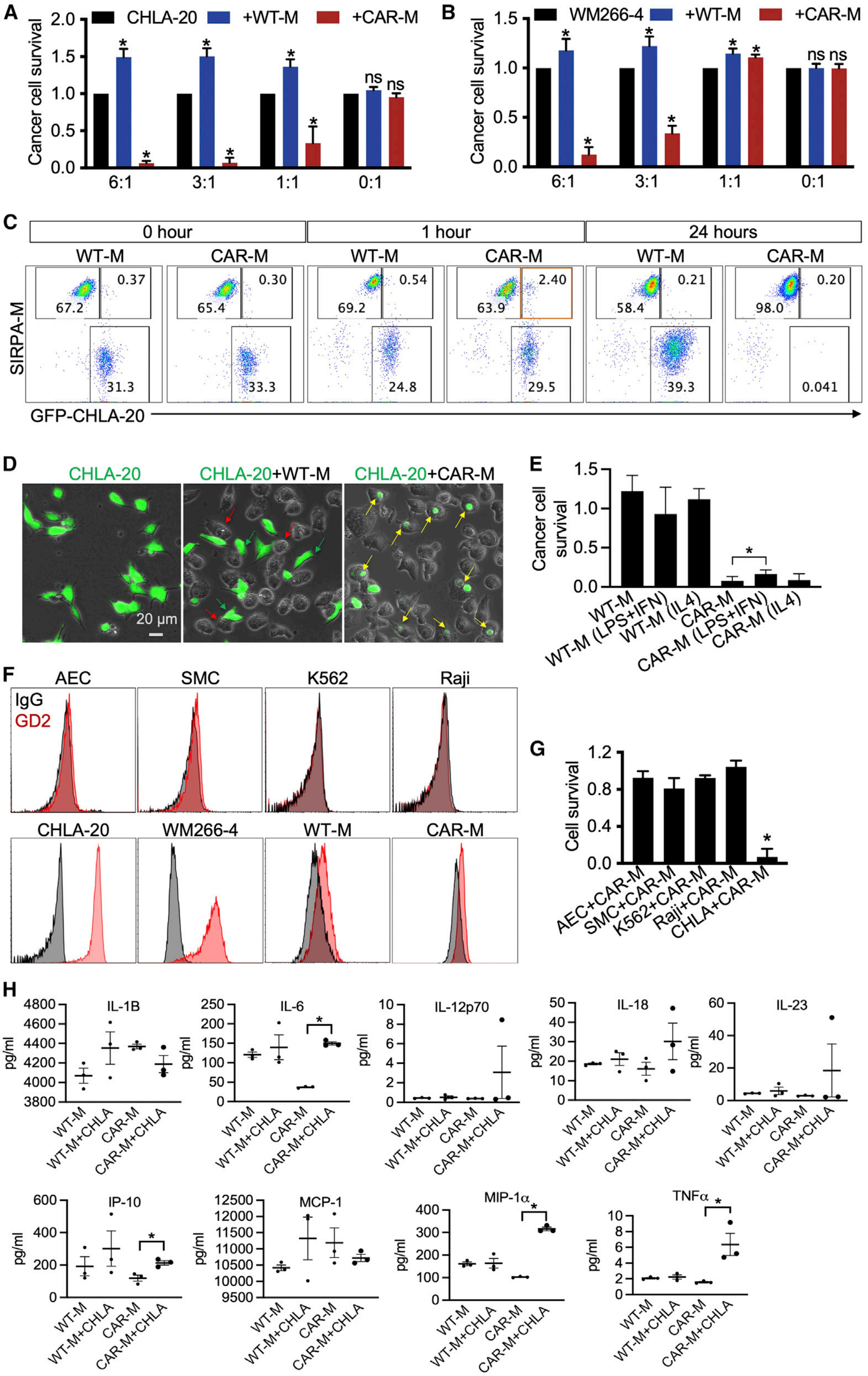

(legend on next page)

CAR cell line from PBMC-3-1 human induced PSCs (hiPSCs) (Figures S1A–S1C) by using the same strategy shown in Figure 3. Anti-GD2 CAR hiPSCs differentiated into GD2-CAR-Ms and demonstrated antitumor activity against both CHLA-20 and WM266-4 cells (Figures S1D and S1E).

To verify antitumor activity *in vivo*, 5×10^5 CHLA-20-AkaLuc-GFP cells were subcutaneously injected into the hind flank of NSG mice alone or with 2.5×10^6 WT-M or CAR-M (H9 hPSC derived) (5:1 E:T ratio) (Figure 5A). These studies revealed that CAR-M-treated mice had significantly reduced tumor burden 4 weeks post injection as evidenced by 90% tumor-free mice with CAR-M treatment compared with 50% tumor-free mice with WT-M treatment (Figure 5B). Significant differences in tumor burden were observed between mice injected with CHLA-20 alone versus mice injected with both CHLA-20 and CAR-Ms, while no statistical differences in tumor burden were observed between mice injected with both CHLA-20 and WT-Ms versus mice with CHLA-20 alone (Figure 5C). CAR-M treatment did not reduce the body weight (Figure 5D), indicating minimal adverse effects of the cell therapy. H&E staining further showed the normal histology of heart, lung, liver, and spleen after CAR-M treatment (Figure S2).

We also tested the antitumor activity of hiPSC-derived CAR-Ms in mice injected with 1×10^6 CHLA-20 and 4×10^6 CAR-M (PBMC-3-1 hiPSC-derived) (4:1 E:T ratio). As shown in Figure S3, hiPSC-derived CAR-Ms significantly reduced tumor burden, while unmodified WT-Ms did not affect tumor growth. In summary, CAR-Ms generated from arterial HE demonstrated superior antitumor activity compared with WT-Ms.

DISCUSSION

Adoptive immunotherapies provide a promising therapeutic option for treating hematologic malignancies. CAR-T and CAR-NK cells represent frontrunners in this approach. Recently, macrophages were investigated for immunotherapy, and the administration of macrophages in patients with cancer has been proved to be well tolerated (Monnet et al., 2002). However, unmodified macrophages exhibited little antitumor activity. Thus, genetic modification of macrophages with CARs is a promising method to bolster their antitumor effects (Klichinsky et al., 2020; Zhang et al., 2020). In previous studies, enhanced antitumor activity was demonstrated in murine macrophages engineered to express CARs with FcRγ, Megf10, and CD3ζ signaling units (Morrissey et al., 2018) and human macrophages expressing the CD3ζ signaling unit (Klichinsky et al., 2020). hiPSC-derived macrophages have been engineered to express CD20-CAR with FcγR1 (Senju et al., 2011), CD19, or mesothelin CAR with CD3ζ signaling domains (Zhang et al., 2020), enabling them to destroy leukemic cells or ovarian cancers. However, the efficacy and persistency need to be further improved in hiPSC-derived CAR-Ms (Zhang et al., 2020).

Here, we demonstrated the utility of a scalable hPSC platform that reproducibly generates uniform CAR-Ms with anti-GD2-dependent antitumor activities through arterial HE. hPSC-derived anti-GD2 CAR-Ms demonstrated superior killing of neuroblastoma and melanoma cells *in vitro* and killing of neuroblastoma cells *in vivo*. To our knowledge, these data are the first evidence of CAR-Ms, hPSC or somatic cell derived, having *in vivo* activity against a pediatric solid tumor.

Figure 4. Evaluation of antitumor activity of CAR-Ms (H9 derived) *in vitro*

(A) Killing of CHLA-20 neuroblastoma cells by CAR-Ms. CHLA-20-AkaLuc-GFP cells were mono-cultured or co-cultured with macrophages at different E:T ratios for 20–24 h. Statistics of CHLA-20 cell survival results are represented as mean ± SD. Student's t test; $*p < 0.05$; ns, not significant; $n > 4$ independent experiments.

(B) Killing of WM266-4 melanoma cells by CAR-Ms. WM266-4-AkaLuc-GFP melanoma cells were mono-cultured or co-cultured with macrophages at different E:T ratios for 20–24 h. Statistics of WM266-4 cell survival. Results are mean ± SD. Student's t test; $*p < 0.05$; ns, not significant; $n > 4$ independent experiments.

(C) Representative flow cytometry plots show CHLA-20 cells co-cultured with WT-Ms and CAR-Ms. CHLA-20 cells are labeled by GFP. WT-Ms and CAR-Ms are labeled by SIRPA immunostaining.

(D) Representative images show phagocytosis of CHLA-20 cells by CAR-Ms. CHLA-20-AkaLuc-GFP cells were co-cultured with macrophages for 6 h (E:T = 3:1). Green arrows indicate CHLA-20 cells, red arrows indicate WT-Ms, and yellow arrows indicate phagocytosis of CAR-Ms.

(E) Killing of CHLA-20 neuroblastoma cells by CAR-Ms with or without treatment. CHLA-20-AkaLuc-GFP cells were mono-cultured or co-cultured with macrophages at E:T ratio = 3:1 for 20–24 h. Statistics of CHLA-20 cell survival results are represented as mean ± SD. Student's t test; $*p < 0.05$; $n = 3$ independent experiments.

(F) Representative histograms show flow cytometry analysis of GD2 expression in different cells. AEC is aretrial endothelial cells, SMC is smooth muscle cells.

(G) CAR-Ms were co-cultured with AEC-NOS3-NanoLuc-2A-tdTomato, SMC-MYH11-NanoLuc-2A-tdTomato, K562-AkaLuc-GFP, Raji-AkaLuc-GFP, and CHLA-20 cells (E:T = 3:1) for 20 h. Target cell survival was measured by luciferase assay. Results are represented as mean ± SD. Student's t test; $*p < 0.05$; $n = 3$ independent experiments.

(H) Secretome analysis of macrophages. WT-Ms and CAR-Ms were mono-cultured or co-cultured with CHLA-20 for 20 h. Cell culture media were collected for secretome analysis. Results are mean ± SD. Student's t test; $*p < 0.05$; $n = 3$ independent experiments.

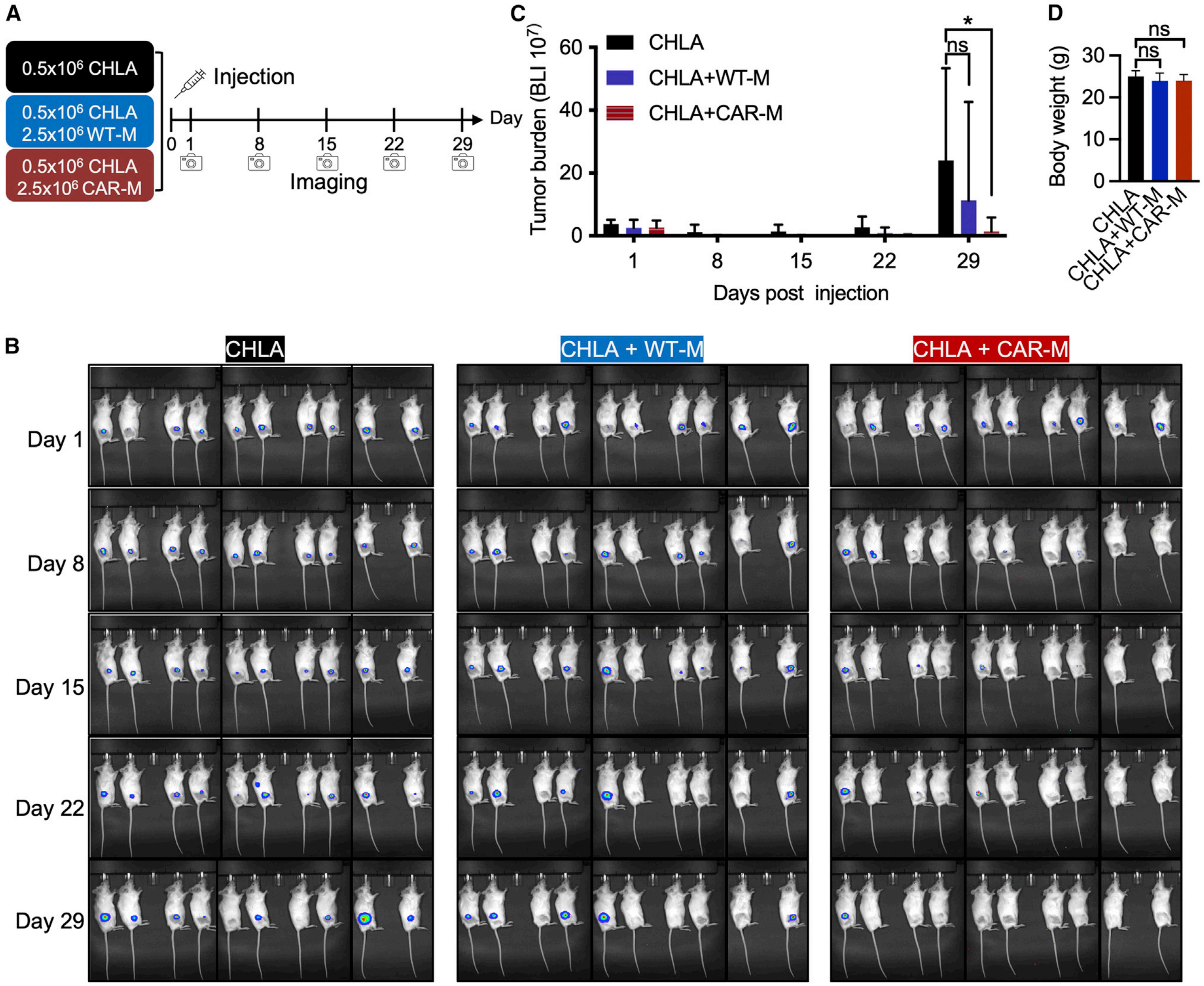

Figure 5. CAR-Ms (H9 derived) significantly reduce tumor burden in a CHLA-20 xenograft mouse model

(A) Schematic of mouse model experiments. CHLA-20-AkaLuc-GFP cells were injected subcutaneously alone or with WT-Ms or CAR-Ms. Luminescent signals were measured at 1, 8, 15, 22, and 29 days post injection. 10 mice per group.

(B) Tumor burden was assessed by bioluminescent imaging at indicated time points.

(C) Quantification of tumor burden as shown by luminescent signals. Results are represented as mean ± SD. Student's t test; $*p < 0.05$; ns, not significant; $n = 10$ mice.

(D) Body weight represented as mean ± SD. Student's t test; ns, not significant; $n = 10$ mice.

hPSC-based platforms for CAR-M production have several advantages over somatic myeloid cell-based platforms. Given that human hPSCs can be expanded almost indefinitely in culture, essentially an unlimited number of the desired therapeutic cells can be produced from an established hPSC line. Additionally, hPSCs can be subjected to multiplex editing approaches to introduce multiple genetic traits and clonally selected to ensure homogeneity of genetic editing. Such uniformity in multiplex gene editing would be difficult to achieve using primary somatic myeloid cells. hPSCs bearing anti-GD2 CARs can be further genetically modified to abrogate human leukocyte antigen (HLA) expression to provide hypoimmunogenic macrophages suitable for administration across HLA immunological barriers. In addition to CAR-Ms, the same gene-edited hPSC cell line could be used for generation of CAR-T or CAR-NK cells for combination immunotherapy.

In prior studies, CAR-Ms were generated via transduction with CAR-expressing lentiviruses (Zhang et al., 2020) or transfection with CAR-expressing plasmids (Senju et al.,

2011). In the present study, we demonstrate that a CAR inserted into the safe-harbor AAVS1 locus in hPSCs by CRISPR-Cas9 avoids the gene silencing that commonly occurs with the above-mentioned methods following differentiation. Overcoming this obstacle in our platform allows for the stable CAR expression in hPSC-derived macrophages. Additionally, we develop an efficient serum- and feeder-free system for macrophage generation based on a modification of our previously established arterial endothelial differentiation protocol. Our anti-GD2 CAR-Ms demonstrate superior antitumor activities against neuroblastoma cells *in vitro* and *in vivo*. Neuroblastoma is the most common and difficult-to-treat type of pediatric solid tumor due to its high genetic heterogeneity (Boeva et al., 2017), making identification of "druggable" targets for small-molecule inhibitors difficult and generation of neoantigens for T cell-based immunotherapies ineffective. However, the neuroblastoma tumor microenvironment is enriched with macrophages (Frosch et al., 2021), and recent data indicate that macrophages could be important mediators of antitumor responses against neuroblastoma in the context of anti-GD2 (redirecting against the tumor) and anti-CD47 (blocking phagocytosis inhibitory pathway) dual-antibody therapy (Theruvath et al., 2022). Here, we show that CAR-Ms may provide an alternative antigen-specific cellular treatment for neuroblastoma. Having an established hPSC-based CAR-M therapy for solid tumors, we are able to explore next-generation cell therapy by introducing additional genetic modifications to enhance antitumor effects or by using a combination CAR-Ms and CAR-T or CAR-NK cell therapies.

EXPERIMENTAL PROCEDURES

Resource availability

Corresponding author

Further information and requests for resources and reagents should be directed to and will be fulfilled by the corresponding author, Dr. Igor I. Slukvin (islukvin@wisc.edu).

Materials availability

Anti-GD2 CAR plasmid and cell lines generated in this study will be made available on request, but we may require a payment and/or a completed materials transfer agreement if there is potential for commercial application.

Data and code availability

NA sequencing (RNA-seq) data is available on Gene Expression Omnibus (GEO) database: GSE201174.

Macrophage differentiation

hPSCs were seeded at low density and differentiated into hematopoietic progenitors by using arterial endothelial cell differentiation media. The hematopoietic progenitors were further induced into macrophages by treatment with macrophage differentiation media.

In vitro cytotoxicity assay

Macrophages were co-cultured with different cells. The target cell survival was measured by luciferase assay.

In vivo antitumor activity analysis

The animal experiments were performed under approval from the UW–Madison Institutional Review Board. Neuroblastoma cells CHLA-20-AkaLuc-GFP and macrophages were injected into the hind flank of the mice. Antitumor effect was monitored by bioluminescent imaging using IVIS imaging system at the indicated time.

Please refer to the supplemental experimental procedures for more detailed methods.

Ethics approval

The animal experiments were performed under approval from UW–Madison, Institutional Review Board.

SUPPLEMENTAL INFORMATION

Supplemental information can be found online at https://doi.org/10.1016/j.stemcr.2022.12.012.

AUTHOR CONTRIBUTIONS

J.Z. designed and performed the experiments, analyzed the data, and wrote the paper. S.W. and B.D. performed cell culture. M. B., J.S., S.S., and R.S. analyzed RNA-seq data. M.F. performed secretome analysis. J.B. performed RNA-seq. M.B. revised the manuscript. A.M. performed cytospin experiment. C.M.C., J.A.T., and I.I.S. designed the experiments and revised the manuscript.

ACKNOWLEDGMENTS

We thank Amy Freitag for editorial assistance. Thanks to Malcolm Brenner (Baylor College of Medicine) for sharing the anti-GD2 CAR sequence. Thanks to Ashley M. Weichmann and Justin Jeffery (Small Animal Imaging & Radiotherapy Facility, UW–Madison) for cell injection and bioluminescent imaging. This work was supported by National Heart, Lung, and Blood Institute grant U01HL134655, R01HL142665, and Cancer Center support grant NIC P30 CA014520 for the University of Wisconsin Small Animal Imaging & Radiotherapy Facility.

CONFLICT OF INTERESTS

C.M.C. receives honorarium for advisory board membership for Elephas Bio, Nektar Therapeutics, and Novartis. I.I.S serves on scientific advisory board for Umoja Biopharma. WARF has filed patent applications on the basis of this work, on which J.Z., A.M.., J.A.T., and I.I.S. are listed as inventors.

Received: August 30, 2022
Revised: December 14, 2022
Accepted: December 14, 2022
Published: January 12, 2023

REFERENCES

Ahmed, N., Brawley, V., Hegde, M., Bielamowicz, K., Kalra, M., Landi, D., Robertson, C., Gray, T.L., Diouf, O., Wakefield, A., et al. (2017). HER2-Specific chimeric antigen receptor-modified virus-specific T cells for progressive glioblastoma: a phase 1 dose-escalation trial. JAMA Oncol. *3*, 1094–1101. https://doi.org/10.1001/jamaoncol.2017.0184.

Ahmed, N., Brawley, V.S., Hegde, M., Robertson, C., Ghazi, A., Gerken, C., Liu, E., Dakhova, O., Ashoori, A., Corder, A., et al. (2015). Human epidermal growth factor receptor 2 (HER2) -specific chimeric antigen receptor-modified T cells for the immunotherapy of HER2-positive sarcoma. J. Clin. Oncol. *33*, 1688–1696. https://doi.org/10.1200/JCO.2014.58.0225.

Alcantara, M., Tesio, M., June, C.H., and Houot, R. (2018). CAR T-cells for T-cell malignancies: challenges in distinguishing between therapeutic, normal, and neoplastic T-cells. Leukemia *32*, 2307–2315. https://doi.org/10.1038/s41375-018-0285-8.

Boeva, V., Louis-Brennetot, C., Peltier, A., Durand, S., Pierre-Eugène, C., Raynal, V., Etchevers, H.C., Thomas, S., Lermine, A., Daudigeos-Dubus, E., et al. (2017). Heterogeneity of neuroblastoma cell identity defined by transcriptional circuitries. Nat. Genet. *49*, 1408–1413. https://doi.org/10.1038/ng.3921.

Brown, C.E., Alizadeh, D., Starr, R., Weng, L., Wagner, J.R., Naranjo, A., Ostberg, J.R., Blanchard, M.S., Kilpatrick, J., Simpson, J., et al. (2016). Regression of glioblastoma after chimeric antigen receptor T-cell therapy. N. Engl. J. Med. *375*, 2561–2569. https://doi.org/10.1056/NEJMoa1610497.

Cheung, I.Y., Cheung, N.K.V., Modak, S., Mauguen, A., Feng, Y., Basu, E., Roberts, S.S., Ragupathi, G., and Kushner, B.H. (2021). Survival impact of anti-GD2 antibody response in a phase II ganglioside vaccine trial among patients with high-risk neuroblastoma with prior disease progression. J. Clin. Oncol. *39*, 215–226. https://doi.org/10.1200/Jco.20.01892.

Frosch, J., Leontari, I., and Anderson, J. (2021). Combined effects of myeloid cells in the neuroblastoma tumor microenvironment. Cancers *13*, 1743. https://doi.org/10.3390/cancers13071743.

Guedan, S., Ruella, M., and June, C.H. (2019). Emerging cellular therapies for cancer. Annu. Rev. Immunol. *37*, 145–171. https://doi.org/10.1146/annurev-immunol-042718-041407.

Heczey, A., Courtney, A.N., Montalbano, A., Robinson, S., Liu, K., Li, M., Ghatwai, N., Dakhova, O., Liu, B., Raveh-Sadka, T., et al. (2020). Anti-GD2 CAR-NKT cells in patients with relapsed or refractory neuroblastoma: an interim analysis. Nat. Med. *26*, 1686–1690. https://doi.org/10.1038/s41591-020-1074-2.

Hegde, M., DeRenzo, C.C., Zhang, H., Mata, M., Gerken, C., Shree, A., Yi, Z., Brawley, V., Dakhova, O., Wu, M.F., et al. (2017). Expansion of HER2-CAR T cells after lymphodepletion and clinical responses in patients with advanced sarcoma. J. Clin. Oncol. *35*, 10508. https://doi.org/10.1200/JCO.2017.35.15_suppl.10508.

Jung, H.S., Uenishi, G., Park, M.A., Liu, P., Suknuntha, K., Raymond, M., Choi, Y.J., Thomson, J.A., Ong, I.M., and Slukvin, I.I. (2021). SOX17 integrates HOXA and arterial programs in hemogenic endothelium to drive definitive lympho-myeloid hematopoiesis. Cell Rep. *34*, 108758. https://doi.org/10.1016/j.celrep.2021.108758.

Klichinsky, M., Ruella, M., Shestova, O., Lu, X.M., Best, A., Zeeman, M., Schmierer, M., Gabrusiewicz, K., Anderson, N.R., Petty, N.E., et al. (2020). Human chimeric antigen receptor macrophages for cancer immunotherapy. Nat. Biotechnol. *38*, 947–953. https://doi.org/10.1038/s41587-020-0462-y.

Lee, Y.G., Chu, H., Lu, Y., Leamon, C.P., Srinivasarao, M., Putt, K.S., and Low, P.S. (2019). Regulation of CAR T cell-mediated cytokine release syndrome-like toxicity using low molecular weight adapters. Nat. Commun. *10*, 2681. https://doi.org/10.1038/s41467-019-10565-7.

Liu, E., Marin, D., Banerjee, P., Macapinlac, H.A., Thompson, P., Basar, R., Nassif Kerbauy, L., Overman, B., Thall, P., Kaplan, M., et al. (2020). Use of CAR-transduced natural killer cells in CD19-positive lymphoid tumors. N. Engl. J. Med. *382*, 545–553. https://doi.org/10.1056/NEJMoa1910607.

Louis, C.U., Savoldo, B., Dotti, G., Pule, M., Yvon, E., Myers, G.D., Rossig, C., Russell, H.V., Diouf, O., Liu, E., et al. (2011). Antitumor activity and long-term fate of chimeric antigen receptor-positive T cells in patients with neuroblastoma. Blood *118*, 6050–6056. https://doi.org/10.1182/blood-2011-05-354449.

Marofi, F., Al-Awad, A.S., Sulaiman Rahman, H., Markov, A., Abdelbasset, W.K., Ivanovna Enina, Y., Mahmoodi, M., Hassanzadeh, A., Yazdanifar, M., Stanley Chartrand, M., and Jarahian, M. (2021a). CAR-NK cell: a new paradigm in tumor immunotherapy. Front. Oncol. *11*, 673276. https://doi.org/10.3389/fonc.2021.673276.

Marofi, F., Motavalli, R., Safonov, V.A., Thangavelu, L., Yumashev, A.V., Alexander, M., Shomali, N., Chartrand, M.S., Pathak, Y., Jarahian, M., et al. (2021b). CAR T cells in solid tumors: challenges and opportunities. Stem Cell Res. Ther. *12*, 81. https://doi.org/10.1186/s13287-020-02128-1.

Matthay, K.K., Maris, J.M., Schleiermacher, G., Nakagawara, A., Mackall, C.L., Diller, L., and Weiss, W.A. (2016). Neuroblastoma. Nat. Rev. Dis. Primers *2*, 16078. https://doi.org/10.1038/nrdp.2016.78.

Monnet, I., Breau, J.L., Moro, D., Lena, H., Eymard, J.C., Ménard, O., Vuillez, J.P., Chokri, M., Romet-Lemonne, J.L., and Lopez, M. (2002). Intrapleural infusion of activated macrophages and gamma-interferon in malignant pleural mesothelioma: a phase II study. Chest *121*, 1921–1927. https://doi.org/10.1378/chest.121.6.1921.

Morrissey, M.A., Williamson, A.P., Steinbach, A.M., Roberts, E.W., Kern, N., Headley, M.B., and Vale, R.D. (2018). Chimeric antigen receptors that trigger phagocytosis. Elife *7*, e36688. https://doi.org/10.7554/eLife.36688.

Norelli, M., Camisa, B., Barbiera, G., Falcone, L., Purevdorj, A., Genua, M., Sanvito, F., Ponzoni, M., Doglioni, C., Cristofori, P., et al. (2018). Monocyte-derived IL-1 and IL-6 are differentially required for cytokine-release syndrome and neurotoxicity due to CAR T cells. Nat. Med. *24*, 739–748. https://doi.org/10.1038/s41591-018-0036-4.

Pan, Y., Yu, Y., Wang, X., and Zhang, T. (2020). Tumor-associated macrophages in tumor immunity. Front. Immunol. *11*, 583084. https://doi.org/10.3389/fimmu.2020.583084.

Pule, M.A., Savoldo, B., Myers, G.D., Rossig, C., Russell, H.V., Dotti, G., Huls, M.H., Liu, E., Gee, A.P., Mei, Z., et al. (2008). Virus-specific

T cells engineered to coexpress tumor-specific receptors: persistence and antitumor activity in individuals with neuroblastoma. Nat. Med. *14*, 1264–1270. https://doi.org/10.1038/nm.1882.

Senju, S., Haruta, M., Matsumura, K., Matsunaga, Y., Fukushima, S., Ikeda, T., Takamatsu, K., Irie, A., and Nishimura, Y. (2011). Generation of dendritic cells and macrophages from human induced pluripotent stem cells aiming at cell therapy. Gene Ther. *18*, 874–883. https://doi.org/10.1038/gt.2011.22.

Switzer, B., Puzanov, I., Skitzki, J.J., Hamad, L., and Ernstoff, M.S. (2022). Managing metastatic melanoma in 2022: a clinical Review. JCO Oncol. Pract. https://doi.org/10.1200/OP.21.00686.

Theruvath, J., Menard, M., Smith, B.A.H., Linde, M.H., Coles, G.L., Dalton, G.N., Wu, W., Kiru, L., Delaidelli, A., Sotillo, E., et al. (2022). Anti-GD2 synergizes with CD47 blockade to mediate tumor eradication. Nat. Med. *28*, 333–344. https://doi.org/10.1038/s41591-021-01625-x.

Uenishi, G.I., Jung, H.S., Kumar, A., Park, M.A., Hadland, B.K., McLeod, E., Raymond, M., Moskvin, O., Zimmerman, C.E., Theisen, D.J., et al. (2018). NOTCH signaling specifies arterial-type definitive hemogenic endothelium from human pluripotent stem cells. Nat. Commun. *9*, 1828. https://doi.org/10.1038/s41467-018-04134-7.

Wang, P.F., Song, S.Y., Wang, T.J., Ji, W.J., Li, S.W., Liu, N., and Yan, C.X. (2018). Prognostic role of pretreatment circulating MDSCs in patients with solid malignancies: a meta-analysis of 40 studies. OncoImmunology *7*, e1494113. ARTN e1494113. https://doi.org/10.1080/2162402X.2018.1494113.

Yu, A.L., Gilman, A.L., Ozkaynak, M.F., London, W.B., Kreissman, S.G., Chen, H.X., Smith, M., Anderson, B., Villablanca, J.G., Matthay, K.K., et al. (2010). Anti-GD2 antibody with GM-CSF, interleukin-2, and isotretinoin for neuroblastoma. N. Engl. J. Med. *363*, 1324–1334. https://doi.org/10.1056/NEJMoa0911123.

Zhang, C., Lv, J., He, Q., Wang, S., Gao, Y., Meng, A., Yang, X., and Liu, F. (2014). Inhibition of endothelial ERK signalling by Smad1/5 is essential for haematopoietic stem cell emergence. Nat. Commun. *5*, 3431. https://doi.org/10.1038/ncomms4431.

Zhang, J., Chu, L.F., Hou, Z., Schwartz, M.P., Hacker, T., Vickerman, V., Swanson, S., Leng, N., Nguyen, B.K., Elwell, A., et al. (2017). Functional characterization of human pluripotent stem cell-derived arterial endothelial cells. Proc. Natl. Acad. Sci. USA *114*, E6072–E6078. https://doi.org/10.1073/pnas.1702295114.

Zhang, L., Tian, L., Dai, X., Yu, H., Wang, J., Lei, A., Zhu, M., Xu, J., Zhao, W., Zhu, Y., et al. (2020). Pluripotent stem cell-derived CAR-macrophage cells with antigen-dependent anti-cancer cell functions. J. Hematol. Oncol. *13*, 153. https://doi.org/10.1186/s13045-020-00983-2.

Stem Cell Reports, Volume *18*

Supplemental Information

Generation of anti-GD2 CAR macrophages from human pluripotent stem cells for cancer immunotherapies

Jue Zhang, Sarah Webster, Bret Duffin, Matthew N. Bernstein, John Steill, Scott Swanson, Matthew H. Forsberg, Jennifer Bolin, Matthew E. Brown, Aditi Majumder, Christian M. Capitini, Ron Stewart, James A. Thomson, and Igor I. Slukvin

Figure legends

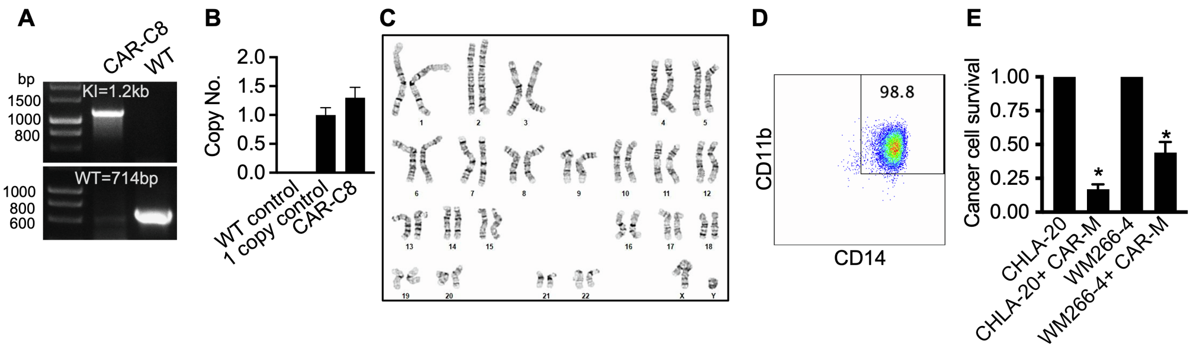

Figure S1. Generation of CAR-M from PBMC-3-1 hiPSCs. Related to Figure 3 and 4.

(A) Junctional PCR analysis AAVS1-CAR KI and WT allele to demonstrate a correct CAR integration.

(B) qPCR analysis of AAVS1-anti-GD2-CAR-PuroR copy number. Data are represented as mean ±SD; n = 3 independent experiments.

(C) Karyotyping of Anti-GD2 CAR hiPSCs.

(D) Representative dot plot shows flow cytometry analysis of CD14 and CD11b expression at day 21 of differentiation.

(E) Killing of CHLA-20 neuroblastoma and WM266-4 melanoma cells by anti-GD2 CAR-M. Cancer cells were mono-cultured or co-cultured with macrophages at E:T ratio=4:1 for 20-24 hours. Statistics of cancer cell survival. Results are mean ±SD. Student's t-test; *, $p < 0.05$; n = 3 independent experiments.

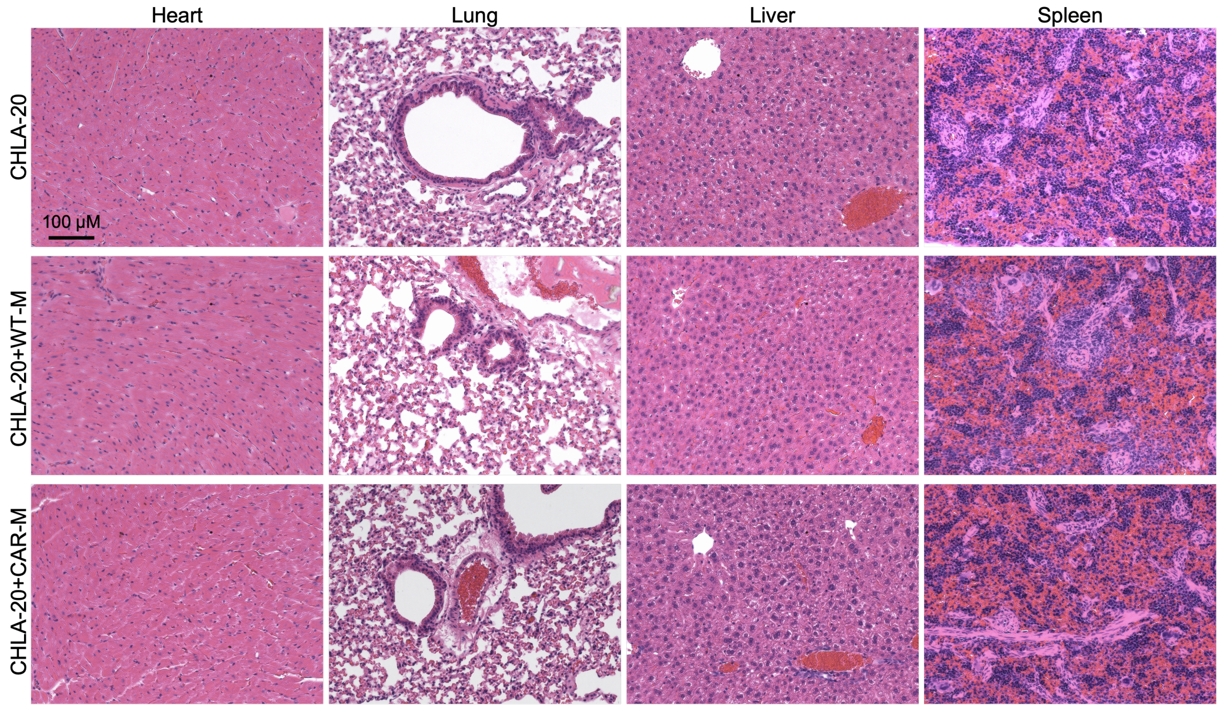

Figure S2. Histology of major organs. Related to Figure 5.

CHLA-20 cells (1x10^6 cells/mouse) were injected subcutaneously. Three days later, WT-M or CAR-M was injected through tail vein (5x10^6 cells/mouse). The mice were sacrificed 5 days later, and the organs were collected for H&E staining.

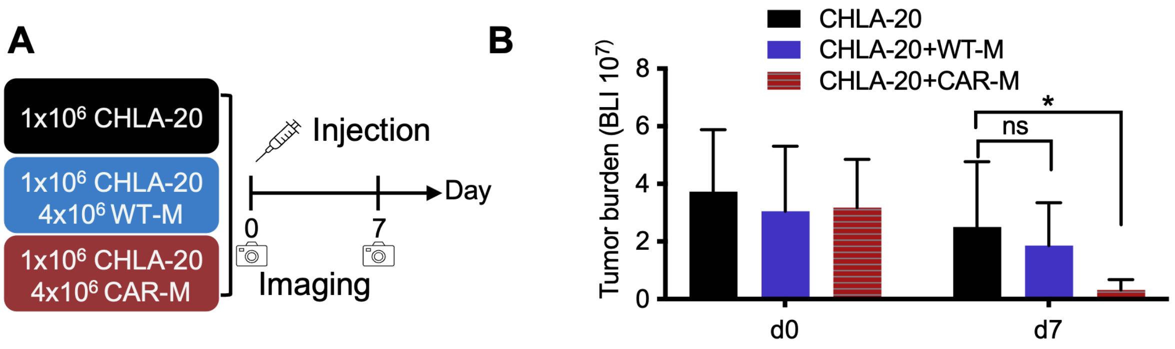

Figure S3. ***In vivo*** **antitumor activity of CAR-M derived from PBMC-3-1 hiPSCs. Related to Figure 5.**

(A) Schematic of mouse model experiments. CHLA-20-AkaLuc-GFP cells were injected alone or with WT-M or CAR-M (derived from PBMC-3-1 hiPSCs) into the hind flank of mice. Luminescent signals were measured 3 hours and 7 days post injection. 8–10 mice per group.

(B) Statistics data of tumor burden was shown by luminescent signals. Data are represented as mean ±SD. Student's t-test; *, $p < 0.05$; n = 8-10 mice.

Table S1. List of Abbreviations

CAR	Chimeric antigen receptor
CAR-M	CAR macrophage
WT-M	Wild type macrophages
PSCs	Pluripotent stem cells
NK cells	Natural killer cells
EHT	Endothelial-to-hematopoietic transition
HE	Hemogenic endothelium
FGF2	Fibroblast growth factor 2
VEGFA	Vascular growth factor A
RESV	Resveratrol
SCF	Stem cell factor
TPO	Thrombopoietin
IL-1B	Interleukin 1 beta
IL3	Interleukin 3
IL6	Interleukin 6
GM-CSF	Granulocyte-macrophage colony-stimulating factor
M-CSF	Macrophage colony-stimulating factor
IFNg	Interferon gamma
LPS	Lipopolysaccharides
GO	Gene ontology
DE	Differential expression
E:T	Effector to target
KOSR	Knockout serum replacement
FBS	Fetal bovine serum
DMSO	Dimethyl sulfoxide

Table S2 Medium components

Medium components	E8	E8BAC	E6	Five factors	FVR	SIT	M4	M5	M36	Vendor	Cat#
DF3S	+	+	+	+	+	+	+	+	+	Thermo Fisher	N/A[a]
Transferrin (10.7 μg/ml)	+	+	+	+	+	+	+	+	+	Fisher Scientific	2914-HT-001G
Insulin (20 μg/ml)	+	+	+		+	+	+	+	+	Sigma	I9287-5ML
FGF2 (100 ng/ml)	+	+		+	+					Homemade	
TGFβ1 (1.7 ng/ml)	+	+								R&D Systems	240-B
BMP4 (5 ng/ml)		+								R&D Systems	314-BP
Activin A (25 ng/ml)		+								R&D Systems	338-AC
CHIR99021 (1 μM)		+								R&D Systems	4423
VEGFA165 (50 ng/ml)				+	+					R&D Systems	293-VE
SB431542 (10 μM)				+						R&D Systems	1614
RESV (5 μM)				+	+					R&D Systems	1418
L690 (10 μM)				+						R&D Systems	0681
SCF (50 ng/ml)						+				R&D Systems	7466-SC
IL3 (10-50 ng/ml)						+			+	Peprotech	200-03
TPO (50 ng/ml)						+				Peprotech	300-18
GM-CSF(200 ng/ml)							+			Peprotech	300-03
IL-1B (10-50 ng/ml)								+		Peprotech	200-01B
M-CSF(20-100 ng/ml)								+	+	Peprotech	300-25
IL-6 (20 ng/ml)									+	Peprotech	200-06
KOSR[b] (10%)								+	+	Fisher Scientific	10828028

[a] DF3S is a customized order which contains DMEM/F12, L-ascorbic acid-2-phosphate magnesium (64 ng/ml), Sodium selenium (14 ng/ml), and $NaHCO_3$ (543 μg/ml).
[b]KOSR can be replace by FBS.

Table S3. PCR primers or probes

Primers/probes	Sequence
J507	TCGACTTCCCCTCTTCCGATG
J508	GAGCCTAGGGCCGGGATTCTC
J487	GACAGCATGTTTGCTGCCTC
J488	CCTGGTGAACACCTAGGACG
PuroR FWD Set 1	GTC ACC GAG CTG CAA GAA
PuroR REV Set 1	CCG ATC TCG GCG AAC AC
PuroR PRB Set 1	/56-FAM/TCG ACA TCG /ZEN/GCA AGG TGT GGG T/3IABkFQ/
TERT FWD Set 1	GAC CAA GCA CTT CCT CTA CTC
TERT REV Set 1	GGA ACC CAG AAA GAT GGT CTC
TERT PRB Set 1	/56-FAM/AGA GAG CTG /ZEN/AGT AGG AAG GAG GGC /3IABkFQ/
CAR FWD Set 1	GGC TTC TGG TTC CTC ATT CA
CAR REV Set 1	GGT TGT AGC TAG TTC CAC CAT AG
CAR PRB Set 1	/56-FAM/ACT GGG TGA /ZEN/GGC AGA ACA TTG GAA /3IABkFQ/
GAPDH	Assay ID: Hs02786624_g1, Thermofisher

Table S4. Antibodies

Antibody	Vendor	Cat#	Dilution
CD31-Alexa 594	Biolegend	303126	1:100
CD31-PE	BD Bioscience	555446	1:100
CD144-FITC	BD Bioscience	560411	1:100
CXCR4-APC	BD Bioscience	560936	1:100
DLL4-APC	Miltenyi	130-096-560	1:100
CD34-APC	BD Bioscience	555824	1:100
CD34-PE	BD Bioscience	560941	1:100
CD45-FITC	BD Bioscience	560976	1:100
CD14-Alexa 488	BD Bioscience	562689	1:100
CD11b-PE	Thermo Fisher	12-0118-42	1:100
Anti-GD2-CAR	Absolute Antibody	Ab02227-1.1	1:200
Anti-mouse IgG	Thermo Fisher	A31571	1:500
CD80-FITC	Fisher Scientific	305205	1:100
CD86-PE	Fisher Scientific	374205	1:100
CD163-PE/Cy7	Fisher Scientific	333613	1:100
CD206-APC	Fisher Scientific	321109	1:100
CD68-FITC	Thermo Fisher	11-0689-42	1:100
SIRPA/CD172A-PE	Biolegend	323806	1:100
GD2-FITC	Fisher Scientific	50-207-7653	1:900
CD90-PE	Biolegend	328109	1:100
CD49f-Alexa 647	BD Bioscience	561557	1:100
CD43-FITC	BD Bioscience	555475	1:100

Supplemental experimental procedures

Statistical analysis

Statistical analysis was performed with two-tailed unpaired Student's t tests as indicated in the figure legends. Data are represented as mean ±SD. A p value of <0.05 was considered to indicate statistical significance.

Maintenance of human pluripotent stem cells (hPSCs)

The experiments were performed under approval from UW–Madison Institutional Review Board. H1 and H9 embryonic stem cells and PBMC-3-1 induced pluripotent stem cells were cultured in E8 medium (Thermo Fisher customized DF3S base medium supplemented with 100 ng/ml FGF2, 1.7 ng/ml TGF-β1, 20 μg/ml insulin, and 10.7 μg/ml Transferrin) on a Matrigel-coated plate (9 μg/cm^2 or 500 μg/plate) (BD Biosciences, Cat # 354230, Batch 2104930). Refer to table 2 for more information.

CAR construct and generation of anti-GD2 CAR-PSCs.

Anti-GD2 CAR (Louis et al., 2011) was cloned into AAVS1-DEST (Oceguera-Yanez et al., 2016) vector and integrated into AAVS1 alleles using CRSPR-Cas9 as previously described (Oceguera-Yanez *et al.*, 2016). CAR integration into AAVS1 locus is confirmed by genomic PCR with the primers (J507/J508 for KI alleles and J487/J488 for WT alleles) listed in table 3.

Copy No. of AAVS1-GD2-CAR-PuroR was determined by qPCR. Genomic DNA was isolated from anti-GD2 CAR PSCs, WT PSCs, and a PCS reporter cell line with one copy

of PuroR cassette. qPCR was performed on ViiA 7 system (Life Technologies) by using the PuroR and TERT probe sets listed in table 3.

CAR sequence

ATGGAGTTTGGGCTGAGCTGGCTTTTTCTTGTGGCTATTTTAAAAGGTGTCCAGTGCTCTAGAGATAT
TTTGCTGACCCAAACTCCACTCTCCCTGCCTGTCAGTCTTGGAGATCAAGCCTCCATCTCTTGCAGAT
CTAGTCAGAGTCTTGTACACCGTAATGGAAACACCTATTTACATTGGTACCTGCAGAAGCCAGGCCA
GTCTCCAAAGCTCCTGATTCACAAAGTTTCCAACCGATTTTCTGGGGTCCCAGACAGGTTCAGTGGC
AGTGGATCAGGGACAGATTTCACACTCAAGATCAGCAGAGTGGAGGCTGAGGATCTGGGAGTTTATT
TCTGTTCTCAAAGTACACATGTTCCTCCGCTCACGTTCGGTGCTGGGACCAAGCTGGAGCTGAAACG
GGCTGATGCTGCACCAACTGTATCCATCTTCCCAGGCTCGGGCGGTGGTGGGTCGGGTGGCGAGG
TGAAGCTTCAGCAGTCTGGACCTAGCCTGGTGGAGCCTGGCGCTTCAGTGATGATATCCTGCAAGG
CTTCTGGTTCCTCATTCACTGGCTACAACATGAACTGGGTGAGGCAGAACATTGGAAAGAGCCTTGA
ATGGATTGGAGCTATTGATCCTTACTATGGTGGAACTAGCTACAACCAGAAGTTCAAGGGCAGGGCC
ACATTGACTGTAGACAAATCGTCCAGCACAGCCTACATGCACCTCAAGAGCCTGACATCTGAGGACT
CTGCAGTCTATTACTGTGTAAGCGGAATGGAGTACTGGGGTCAAGGAACCTCAGTCACCGTCTCCTC
AGCCAAAACGACACCCCCATCAGTCTATGGAAGGGTCACCGTCTCTTCAGCGGAGCCCAAATCTTGT
GACAAAACTCACACATGCCCACCGTGCCCGGATCCCAAATTTTGGGTGCTGGTGGTGGTTGGTGGA
GTCCTGGCTTGCTATAGCTTGCTAGTAACAGTGGCCTTTATTATTTTCTGGGTGAGGAGTAAGAGGA
GCAGGCTCCTGCACAGTGACTACATGAACATGACTCCCCGCCGCCCCGGGCCCACCCGCAAGCATT
ACCAGCCCTATGCCCCACCACGCGACTTCGCAGCCTATCGCTCCAGGGACCAGAGGCTGCCCCCC
GATGCCCACAAGCCCCCTGGGGGAGGCAGTTTCCGGACCCCCATCCAAGAGGAGCAGGCCGACGC
CCACTCCACCCTGGCCAAGATCAGAGTGAAGTTCAGCAGGAGCGCAGACGCCCCCGCGTACCAGC
AGGGCCAGAACCAGCTCTATAACGAGCTCAATCTAGGACGAAGAGAGGAGTACGATGTTTTGGACA
AGAGACGTGGCCGGGACCCTGAGATGGGGGGAAAGCCGAGAAGGAAGAACCCTCAGGAAGGCCT
GTACAATGAACTGCAGAAAGATAAGATGGCGGAGGCCTACAGTGAGATTGGGATGAAAGGCGAGCG
CCGGAGGGGCAAGGGGCACGATGGCCTTTACCAGGGTCTCAGTACAGCCACCAAGGACACCTACG
ACGCCCTTCACATGCAGGCCCTGCCCCCTCGCTAA

RT-qPCR of CAR expression

CAR expression was determined by RT-qPCR. Reverse transcription was performed by using SuperScript™ IV VILO™ Master Mix (Life Technologies, Cat # 11756050). qPCR was performed on ViiA 7 system (Life Technologies) by using the CAR and GAPDH probes listed in table 3.

Immunostaining of GD2-CAR

Macrophages were treated with lipopolysaccharides (LPS, 1X) (Thermo Fisher, Cat # 00-4976-03) and interferon gamma (IFNγ, 100 ng/ml) (PeproTech, Cat # 300-02) for 2–4 days to improve the cell attachment during the staining. Macrophages were fixed in cold methanol for 10 mins at -20°C and then blocked and permeabilized with 2.5% donkey serum and 0.2% Triton-X100 for 20 mins at room temperature. Anti-Disialoganglioside GD2 antibody 14G2a (Absolute Antibody, clone 1A7, Cat # Ab02227-1.1, 1:200 dilution) was added and incubated for 2 hours at room temperature. After removing the primary antibody, a secondary antibody (Thermo Fisher, Anti-mouse IgG-Alexa Flour 647, Cat # A31571, 1:500 dilution) was applied and incubated for 1 hour at room temperature. Macrophages were gently washed with PBS between each staining steps. Images were taken by Nikon confocal microscopy.

High density differentiation

The experiments were performed under approval from UW–Madison Institutional Review Board. Human PSCs were cultured in E8 medium on a Matrigel-coated plate (9 μg/cm^2 or 500 μg/plate). To achieve the best differentiation results, PSCs were split using EDTA (0.5 mM in PBS, osmolarity 340 mOsm) (Thermo Fisher, Cat # 1575020) at 1:4 ratio 2 days before differentiation. The cells reached 80–90% confluency 2 days later. At the day of differentiation (day 0), PSCs were dissociated by Accutase (Invitrogen, Cat # A1110501) for ~5 min at 37°C. To induce mesoderm differentiation, the cells were plated on a vitronectin-coated (0.9 μg/cm^2 or 50 μg/plate) (60-478 aa, homemade) plate (1.1 x 10^5 cells/cm^2) by using E8BAC medium for 2 days (usually, 44 hours). To improve cell survival, 10 μM Y27632 (R&D Systems, Cat # 1254) was used at day 0. From day 2 to

day 6, “five factor” medium was used. From day 6 to day 10, FVR medium was used. Please refer to table 2 for more information about the media.

Low density differentiation optimized for generation of hematopoietic progenitors

To induce mesoderm differentiation, PSCs were plated on a vitronectin-coated (0.9 μg/cm^2, or 50 μg/plate) plate (1.8 x 10^4 cells/cm^2) by using E8BAC media for 2 days (usually, 44 hours). To improve cell survival, 10 μM Y27632 was used at day 0. From day 2 to day 6, “five factor” medium was used. From day 6 to day 10, FVR medium was used. The day 10 cells can be used for macrophage differentiation or further expanded in SIT medium for 3 days in order to enhance proportion and yield of CD34$^+$CD45$^+$ progenitors.

From day 0–6: medium (12ml/ plate) was changed every day.

From day 6–10: medium (24 ml/ plate) was changed every other day.

Please refer to table 2 for more information about the media.

The day 10 or day 13 hematopoietic progenitor cells can be cryopreserved in 90% fetal bovine serum (FBS) with 10% dimethyl sulfoxide (DMSO).

An alternative protocol for generation of hematopoietic progenitors

An alternative protocol can be used to further improve hematopoietic progenitor generation. The PSCs were plated on a vitronectin-coated plate at high density (1.1 x 10^5 cells/cm^2) in E8BAC medium for 2 days. The mesoderm cells were passaged and seeded on a new vitronectin-coated plate at low density (about 1:8 split, 0.7-1.1 x 10^5 cells/cm^2). From day 2 to day 6, “five factor” medium was used. From day 6 to day 10, FVR medium was used.

The mesoderm cells can be cryopreserved in E8BAC medium with 10% DMSO, so it saves the time for PSCs culture and mesoderm differentiation.

The hematopoietic progenitor cells can be cryopreserved in 90% fetal bovine serum (FBS) with 10% dimethyl sulfoxide (DMSO).

Please refer to table 2 for more information about the media.

Macrophage differentiation

The round shape hematopoietic progenitor cells were collected from above hematopoietic differentiation protocol on day 10, transferred to low-adhesion plate, and cultured at a concentration of 1 x 10^6 cells/ml in M4 media for 3 days. Three days later, the cells were transferred to 10-cm dish or 6-well plate (non-coating, regular cell culture plate) (1-2 x 10^4 cells/cm^2) with M5 media for another 7–15 days. Half of the M5 was changed every 3–4 days.

A modified protocol can be used to further improve the CAR-M differentiation. The PSCs were plated on a vitronectin-coated plate (1.1 x 10^5 cells/cm^2) in E8BAC medium for 2 days. The mesoderm cells were passaged and seeded on a new vitronectin-coated plate (0.7-1.1 x 10^5 cells/cm^2). From day 2 to day 7, "five factor" media was used. From day 7 to day 11, FVR media was used. The day 11 hematopoietic progenitor cells were transferred to a new plate (non-coating, regular cell culture plate) and cultured in M36 media for 3 days (day 11–14, 1 x 10^6 cells/ml). The cells were them transferred to another new plate (non-coating, regular cell culture plate) (1-2 x 10^4 cells/cm^2) with M5 media for another 7–15 days. Half of the M5 was changed every 4 days.

Please refer to table 2 for more information about the media.

Flow cytometry

Cells (0.1-1 x 10^6) were resuspended in diluted antibodies and incubated at 4°C for 0.5–1 hour. Cells were then washed with 2% FBS-PBS for 1–2 times and cytometric analysis was performed on BD FACSCanto II. FlowJo was used for the data analysis.

Please refer to table 4 for more information about the antibody.

RUNX1-venus reporter cell line

RUNX1+23-Venus reporter cell line was previously generated by Slukvin lab (Uenishi et al., 2018).

Hematopoietic colony forming unit assay

Hematopoietic colony forming unit assay was conducted in serum-containing Methocult (Stem Cell Technologies, Cat # H4436) according to manufacturer instruction. Day 8 cells (both the attached and floating cells) were collected for analysis.

Phagocytosis of BioParticles

Macrophages were seeded on 12-well plate (2 x 10^5 cells/well, non-coating) with M5 medium. Add 100 µl of 1mg/ml Zymosan A BioParticles (2 x 10^7 particles/mg solid) (Thermo Fisher, Cat # Z2843) to each well. Phagocytosis was imaged over a 24 hour time period (image capture every 10–30 minutes) (Nikon, BioCT).

***In vitro* cytotoxicity/phagocytosis assay**

AkaLuc-GFP was cloned into AAVS1-DEST vector and then integrated into AAVS1 locus of CHLA-20, WM266-4, K562, and Raji cells by using CRISPR-Cas9 technology (Oceguera-Yanez *et al.*, 2016). Arterial endothelial cells and smooth muscle cells were derived from NOS3-NanoLuc-tdTomato and MYH11-NanoLuc-tdTomato H1 cell lines, respectively, using previously described protocols ((Zhang et al., 2017; Zhang et al., 2019)). To assess cytotoxicity, cells were incubated at indicated effectors:targets (E:T) ratio of 3:1 for 20–24 hours in 96-well plate. Luciferase substrate (50–250 μM Tokeoni for AkaLuc, R&D Systems, Cat # 6555) (Nano-Glo for NanoLuc, Promega, Cat # N1120, 1:1000 dilution) was added to the cell culture and bioluminescence was measured using Promega GloMax plate reader 15 minutes later.

For imaging, macrophages were seeded in 96-well plate (2-6 x 10^4 cells/well, non-coating) with M5 media with or without CHLA-20 (2 x 10^4 cells/well). Images were taken on EVOS Cell Imaging System (Thermo Fisher). Time-lapse imaging was taken over a 20–24 hour time period (image capture every 20 min) by using BioCT (Nikon).

In vivo anti-tumor activity analysis

The experiments were performed under approval from UW–Madison, Institutional Review Board. NOD-scid IL2Rgamma^{null} (NSG) mice (6 to 8 week old) were obtained from Jackson Lab. Neuroblastoma cells CHLA20-AkaLuc-GFP and macrophages were injected into the hind flank of the mice. Antitumor effect was monitored by bioluminescent imaging using IVIS imaging system at the indicated time (100 μl 5mM Tokeoni/mouse).

Secretome assay

Secretome assay was performed according to the manufacturer's instruction (Mesoscale, U-PLEX Macrophage M1 Combo 1, Cat # K15336K).

Statistical analysis

P value was calculated by Student's t-test, tails = 2, type = 2.

RNA-sequencing

WT-M and CAR-M were co-cultured with CHLA-20 cells (E:T = 3:1) for 1 day as described in "*in vitro* cytotoxicity assay" and then $CD14^{+}CD11b^{+}$ macrophage were sorted by flowcytometry (BD FACSAria). Total RNA was isolated from the co-cultured purified macrophages or mono-cultured macrophages by using the RNeasy Plus Micro Kit (Qiagen, Cat # 74034) and quantified with both the Quant-It RNA Assay Kit (Thermo Fisher, Q10210) and the Bioanalyzer RNA 6000 Pico Kit (Agilent). The cDNA library was prepared using a custom LM-Seq protocol previously described in the previous study(Hou et al., 2015). The final indexed cDNA libraries were pooled with 19 samples per lane and run on the NextSeq 2000 (Illumina).

Read Processing

The sequencer outputs were processed using Illumina's Dragen 3.8.4 BCLConvert software. Adapter sequences and low-quality ends were trimmed from reads. Each sample's reads were then processed using RSEM version 1.2.3 (with bowtie-0.12.9 for the alignment step), aligning to a HG38 reference. RSEM input parameters were specified such that alignments were suppressed for a read if over 200 valid alignments existed ('--

bowtie-m' option), a maximum of two mismatches were allowed in the seed ('-- bowtie-n' option), and the probability of generating a read from the forward strand of the transcript was 0.5 since reads were not strand-specific ('--forwardprob' option).

Differential Expression Analysis

Differentially Expressed genes were determined using DESeq2 v1.28.1, running under R v4.0.

Gene Ontology (GO) Analysis and Heatmaps

The most highly differentially expressed genes were then subjected to GO analysis. A threshold of unadjusted p-val <= 0.05 was used, resulting in 807 and 868 down-regulated and up-regulated genes respectively. Gene set enrichment analysis on the differential expression (DE) genes was performed using GOSeq (Young et al., 2010) using all Biological Process gene sets in the Gene Ontology downloaded from MSigDB (Subramanian et al., 2005). GO ontology terms related to macrophages were chosen for display. For heatmapping, the gene expression data was normalized by computing log(TPM+1) and then for each gene, the Z-score was computed by subtracting the mean and dividing by the standard deviation over all of the samples selected for the heatmap.

References

Hou, Z., Jiang, P., Swanson, S.A., Elwell, A.L., Nguyen, B.K., Bolin, J.M., Stewart, R., and Thomson, J.A. (2015). A cost-effective RNA sequencing protocol for large-scale gene expression studies. Sci Rep *5*, 9570. 10.1038/srep09570.

Louis, C.U., Savoldo, B., Dotti, G., Pule, M., Yvon, E., Myers, G.D., Rossig, C., Russell, H.V., Diouf, O., Liu, E., et al. (2011). Antitumor activity and long-term fate of chimeric antigen receptor-positive T cells in patients with neuroblastoma. Blood *118*, 6050-6056. 10.1182/blood-2011-05-354449.

Oceguera-Yanez, F., Kim, S.I., Matsumoto, T., Tan, G.W., Xiang, L., Hatani, T., Kondo, T., Ikeya, M., Yoshida, Y., Inoue, H., and Woltjen, K. (2016). Engineering the AAVS1 locus for consistent and scalable transgene expression in human iPSCs and their differentiated derivatives. Methods *101*, 43-55. 10.1016/j.ymeth.2015.12.012.

Subramanian, A., Tamayo, P., Mootha, V.K., Mukherjee, S., Ebert, B.L., Gillette, M.A., Paulovich, A., Pomeroy, S.L., Golub, T.R., Lander, E.S., and Mesirov, J.P. (2005). Gene set enrichment analysis: a knowledge-based approach for interpreting genome-wide expression profiles. Proc Natl Acad Sci U S A *102*, 15545-15550. 10.1073/pnas.0506580102.

Uenishi, G.I., Jung, H.S., Kumar, A., Park, M.A., Hadland, B.K., McLeod, E., Raymond, M., Moskvin, O., Zimmerman, C.E., Theisen, D.J., et al. (2018). NOTCH signaling specifies arterial-type definitive hemogenic endothelium from human pluripotent stem cells. Nat Commun *9*, 1828. 10.1038/s41467-018-04134-7.

Young, M.D., Wakefield, M.J., Smyth, G.K., and Oshlack, A. (2010). Gene ontology analysis for RNA-seq: accounting for selection bias. Genome Biol *11*, R14. 10.1186/gb-2010-11-2-r14.

Zhang, J., Chu, L.F., Hou, Z., Schwartz, M.P., Hacker, T., Vickerman, V., Swanson, S., Leng, N., Nguyen, B.K., Elwell, A., et al. (2017). Functional characterization of human pluripotent stem cell-derived arterial endothelial cells. Proc Natl Acad Sci U S A *114*, E6072-E6078. 10.1073/pnas.1702295114.

Zhang, J., McIntosh, B.E., Wang, B., Brown, M.E., Probasco, M.D., Webster, S., Duffin, B., Zhou, Y., Guo, L.W., Burlingham, W.J., et al. (2019). A Human Pluripotent Stem Cell-Based Screen for Smooth Muscle Cell Differentiation and Maturation Identifies Inhibitors of Intimal Hyperplasia. Stem Cell Reports *12*, 1269-1281. 10.1016/j.stemcr.2019.04.013.
